# Square and rhombic lattices of magnetic skyrmions in a centrosymmetric binary compound

**DOI:** 10.1038/s41467-022-29131-9

**Published:** 2022-03-30

**Authors:** Rina Takagi, Naofumi Matsuyama, Victor Ukleev, Le Yu, Jonathan S. White, Sonia Francoual, José R. L. Mardegan, Satoru Hayami, Hiraku Saito, Koji Kaneko, Kazuki Ohishi, Yoshichika Ōnuki, Taka-hisa Arima, Yoshinori Tokura, Taro Nakajima, Shinichiro Seki

**Affiliations:** 1 Department of Applied Physics, University of Tokyo, Tokyo, 113-8656 Japan; 2 Institute of Engineering Innovation, University of Tokyo, Tokyo, 113-0032 Japan; 3 PRESTO, Japan Science and Technology Agency (JST), Kawaguchi, 332-0012 Japan; 4 RIKEN Center for Emergent Matter Science (CEMS), Wako, 351-0198 Japan; 5 Laboratory for Neutron Scattering and Imaging (LNS), Paul Scherrer Institute (PSI), 5232 Villigen, Switzerland; 6 Laboratory for Ultrafast Microscopy and Electron Scattering (LUMES), Institute of Physics, École Polytechnique Fédérale de Lausanne (EPFL), 1015 Lausanne, Switzerland; 7 Laboratory of Nanoscale Magnetic Materials and Magnonics (LMGN), Institute of Materials, École Polytechnique Fédérale de Lausanne (EPFL), 1015 Lausanne, Switzerland; 8 Deutsches Elektronen-Synchrotron DESY, Notkestraße 85, 22607 Hamburg, Germany; 9 The Institute for Solid State Physics, University of Tokyo, Kashiwa, 277-8561 Japan; 10 Materials Sciences Research Center, Japan Atomic Energy Agency, Tokai, 319-1195 Japan; 11 J-PARC Center, Japan Atomic Energy Agency, Tokai, 319-1195 Japan; 12 Neutron Science and Technology Center, Comprehensive Research Organization for Science and Society (CROSS), Tokai, 319-1106 Japan; 13 Department of Advanced Materials Science, University of Tokyo, Kashiwa, 277-8561 Japan; 14 Tokyo College, University of Tokyo, Tokyo, 113-8656 Japan

**Keywords:** Magnetic properties and materials, Spintronics

## Abstract

Magnetic skyrmions are topologically stable swirling spin textures with particle-like character, and have been intensively studied as a candidate of high-density information bit. While magnetic skyrmions were originally discovered in noncentrosymmetric systems with Dzyaloshinskii-Moriya interaction, recently a nanometric skyrmion lattice has also been reported for centrosymmetric rare-earth compounds, such as Gd_2_PdSi_3_ and GdRu_2_Si_2_. For the latter systems, a distinct skyrmion formation mechanism mediated by itinerant electrons has been proposed, and the search of a simpler model system allowing for a better understanding of their intricate magnetic phase diagram is highly demanded. Here, we report the discovery of square and rhombic lattices of nanometric skyrmions in a centrosymmetric binary compound EuAl_4_, by performing small-angle neutron and resonant elastic X-ray scattering experiments. Unlike previously reported centrosymmetric skyrmion-hosting materials, EuAl_4_ shows multiple-step reorientation of the fundamental magnetic modulation vector as a function of magnetic field, probably reflecting a delicate balance of associated itinerant-electron-mediated interactions. The present results demonstrate that a variety of distinctive skyrmion orders can be derived even in a simple centrosymmetric binary compound, which highlights rare-earth intermetallic systems as a promising platform to realize/control the competition of multiple topological magnetic phases in a single material.

## Abstract (web summary)

Typically, skyrmions appear in magnet systems which are non-centrosymmetric. Here, using neutron and X-ray scattering, Takagi et al show the emergence of a skyrmion phase in the centrosymmetric material EuAl_4_. This expands the range of materials potential hosting skyrmions.

## Introduction

**Fig. 1:**
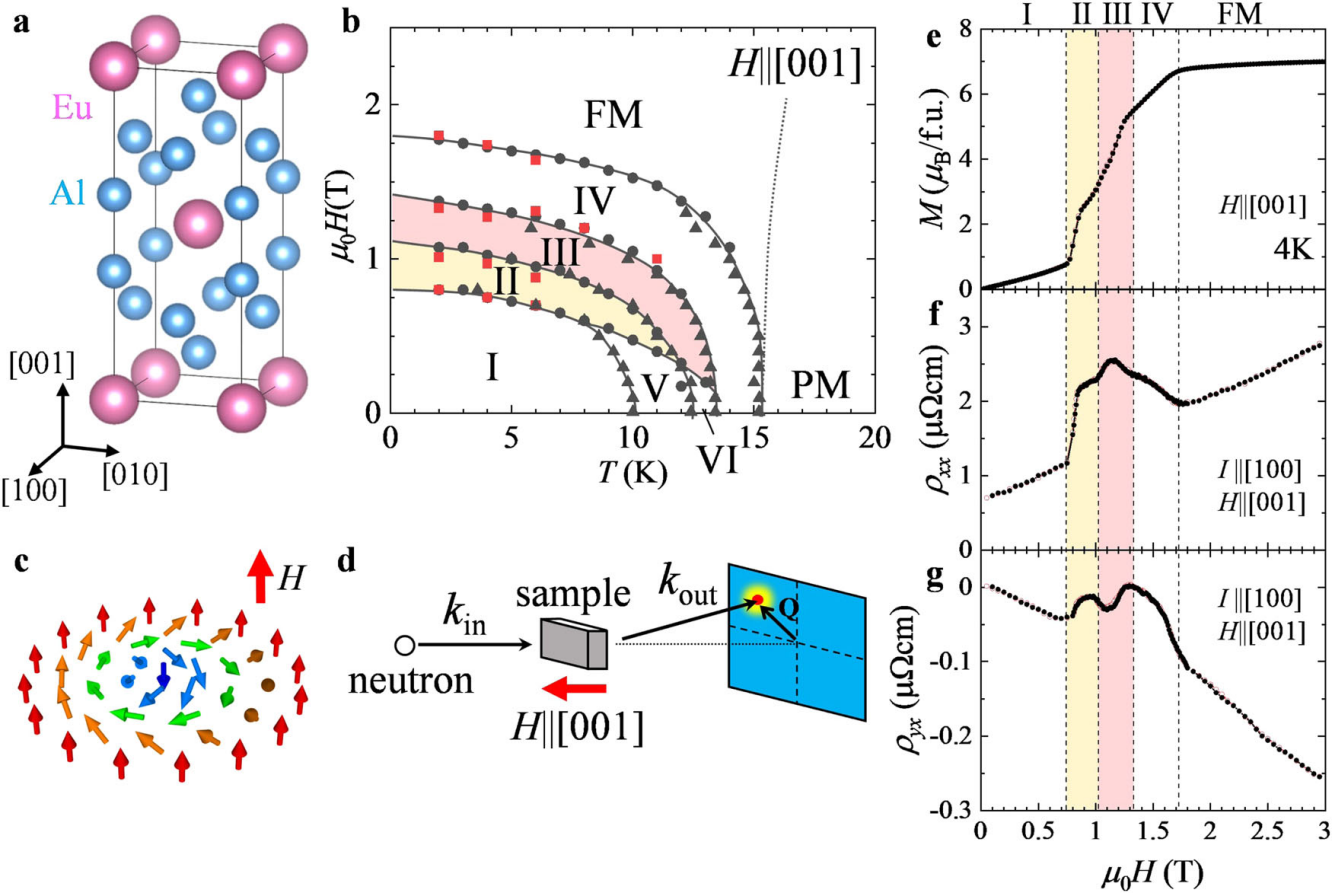
Magnetic phase diagram of EuAl_4_. **a** Crystal structure of EuAl_4_. **b** *H*(magnetic field)-*T*(temperature) phase diagram for *H* || [001] determined from *T* and *H* dependences of magnetization, *M* (black triangular and circle symbols, respectively), and the *H* dependence of Hall resistivity, *ρ*_*yx*_ (red square symbols). FM and PM represent the ferromagnetic and paramagnetic states, respectively. **c** Schematic illustration of the skyrmion spin texture. **d** Schematic illustration of the experimental geometry for the SANS measurement. *k*_in_ and *k*_out_ are the incident and scattered neutron wave vectors, respectively. **e**–**g** Magnetic-field dependence of magnetization *M* (**e**), longitudinal resistivity *ρ*_*xx*_ (**f**), and Hall resistivity *ρ*_*yx*_ (**g**) at 4 K for *H* || [001] and *I* || [100]. Black filled and red open circles correspond to the field-increasing and -decreasing processes, respectively.

Recently, the concept of topology is attracting attention as a source for various exotic phenomena in solids. One typical example is magnetic skyrmion, i.e., a noncoplanar swirling spin structure with topologically stable particle-like nature (Fig. 1c). It is characterized by non-zero integer skyrmion number *N*_sk_ defined as:

1 $${N}_{{{{{{{\mathrm{sk}}}}}}}}=\frac{1}{4\pi }\int {{{{{\bf{n}}}}}}\cdot \left(\frac{\partial {{{{{\bf{n}}}}}}}{\partial x}\times \frac{\partial {{{{{\bf{n}}}}}}}{\partial y}\right){dxdy}$$

with **n**(**r**) = **m(r)/|m(r)**| being the unit vector along the direction of local magnetic moment **m**(**r**), which represents how many times **n**(**r**) wrap a sphere^1^. In metallic compounds, skyrmion spin textures induce fictitious magnetic fluxes acting on the conduction electrons through quantum-mechanical Berry phase, which leads to various exotic transport phenomena, such as the topological Hall effect^2–4^. Conversely, an applied electric current can efficiently drive the skyrmion motion through a spin transfer torque, whose threshold current density is often five orders of magnitude smaller than for conventional ferromagnetic domain walls^3,4^. These features highlight skyrmions as potential novel information carriers with high-energy efficiency and information density.

Previously, magnetic skyrmions were mostly observed in noncentrosymmetric systems, where a competition between the ferromagnetic exchange and Dzyaloshinskii-Moriya (DM) interactions stabilizes a triangular skyrmion lattice (SkL) state^1,5–15^. On the other hand, a SkL has recently been discovered for centrosymmetric rare-earth-based compounds. In the latter systems, the symmetry of the SkL usually reflects that of the underlying crystal lattice, such as triangular SkL in hexagonal Gd_2_PdSi_3_ and Gd_3_Ru_4_Al_12_ and square SkL in tetragonal GdRu_2_Si_2_^16–18^. Notably, these centrosymmetric materials commonly host extremely small skyrmions of diameter less than 5 nm, which is almost one order of magnitude smaller than the typical skyrmion diameter in conventional DM-based bulk magnets. For such systems, a distinct skyrmion formation mechanism mediated by itinerant electrons has been proposed^19–22^, and this theoretical framework successfully explains the magnetic phase diagram and the associated spin-charge coupling in GdRu_2_Si_2_^17,23^. To date, the examples of skyrmion-hosting centrosymmetric magnets are limited to a few Gd-based intermetallics, and further search of simpler model compounds and better understanding of their intricate magnetic phase diagram are highly demanded.

In this work, we focus on a simple centrosymmetric binary compound EuAl_4_, and investigate its magnetic structure by means of neutron and X-ray scattering experiments. Unlike previously reported systems, EuAl_4_ turns out to host two distinctive topological magnetic phases, i.e., a square and a rhombic lattice state of skyrmions with a diameter of 3.5 nm, which is accompanied by the multiple-step reorientation of fundamental magnetic modulation vector as a function of magnetic field. The present results highlight EuAl_4_ as the simple model system to embody the unique itinerant-electron-mediated skyrmion formation mechanism, and suggest that a similar competition among multiple topological magnetic phases can be widely expected in other rare-earth-based intermetallic systems.

## Results

Our target material EuAl_4_ has the centrosymmetric tetragonal crystal structure, which belongs to the space group *I*4/*mmm* (No. 139) as shown in Fig. 1a^24^. This compound is reported to form a charge density wave characterized by the incommensurate ordering vector along the [001] axis below 145 K^25^ (See Supplementary Note X for the detailed discussion on the charge density wave and crystal symmetry). The magnetism is governed by the Eu^2+^ (*S* = 7/2, *L* = 0) ions with Heisenberg magnetic moment, and the square lattice layers of Eu^2+^ and nonmagnetic Al layers are alternately stacked along the [001] axis. According to the previous neutron scattering experiment under zero magnetic field, this compound hosts incommensurate magnetic orders below *T*_N_ = 15.4 K^26^. It is characterized by the weak easy-axis magnetic anisotropy, and shows multiple-step metamagnetic transitions as sweeping the external magnetic field **H** || [001]^24^. Very recently, the non-monotonous *H*-dependence of Hall resistivity was reported, and its possible relevance to topological Hall effect was discussed^27^. At this stage, the magnetic structure for each phase in magnetic fields has not been identified experimentally.

First, we investigated the magnetic phase diagram of EuAl_4_ for **H** || [001]. Figure 1e–g shows the *H*-dependence of magnetization *M*, resistivity *ρ*_*xx*_, and Hall resistivity *ρ*_*yx*_ measured at 4 K for **H** || [001] and **I** || [100], respectively. The magnetization curves show multiple-step metamagnetic transitions before reaching the forced ferromagnetic (FM) state, which is accompanied by non-monotonous changes in *ρ*_*xx*_ and *ρ*_*yx*_. These behaviors are in agreement with previous reports^24,27^ (See Supplementary Note V for the detailed interpretation of *ρ*_*yx*_ profile). By performing similar measurements at selected temperatures, the *H*–*T* (temperature) magnetic phase diagram is summarized in Fig. 1b. Below 8 K, four distinctive magnetic phases (i.e., phases I, II, III, and IV) are identified. The *H-*values for the anomalies observed in the *M*, *ρ*_*xx*_ and *ρ*_*yx*_ profiles coincide with each other, which suggests a strong coupling between the magnetism and the electrical transport properties.

**Fig. 2:**
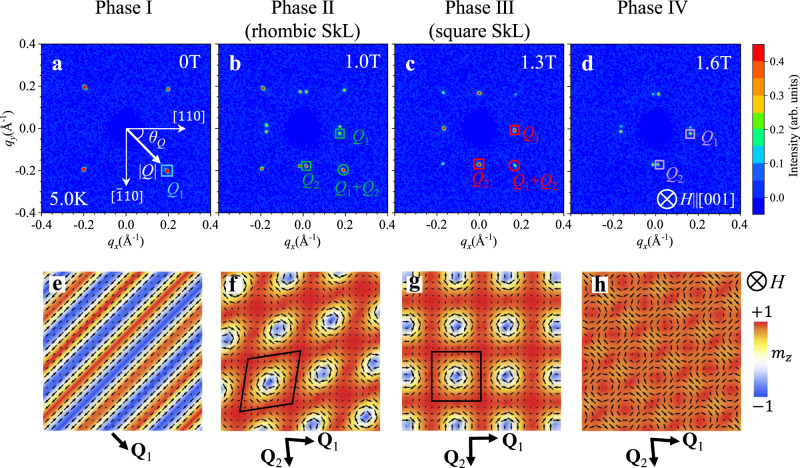
Magnetic-field dependence of SANS patterns for EuAl_4_. **a**–**d** Typical SANS patterns taken at 5.0 K at various strengths of magnetic field for *H* || [001]. The color scale indicates the integrated intensity. **e**–**h** Schematics of the screw (**e**), rhombic skyrmion lattice (**f**), square skyrmion lattice (**g**), and vortex-lattice (**h**) spin textures. Each phase is characterized by distinctive orientation of the fundamental magnetic modulation vectors **Q**_1_ and **Q**_2_. The rhombic and square skyrmion lattice states are the double-*Q* states described by Eq. (2), i.e., the superposition of two obliquely or orthogonally modulated spin helices. Background color represents the out-of-plane component of local magnetic moment *m*_*z*_. See Supplementary Note VII and VIII for the detailed spin texture in phase IV.

**Fig. 3:**
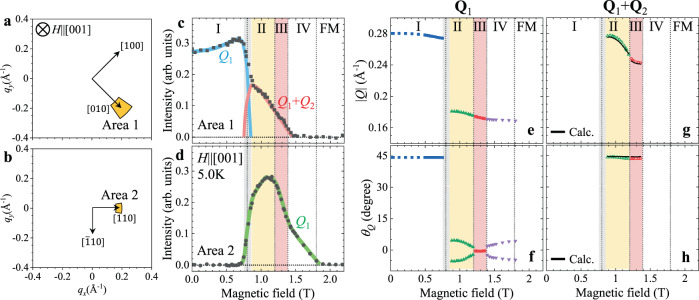
Magnetic-field dependence of the magnetic modulation vectors in EuAl_4_. **a**, **b** Schematic illustrations of Area 1 and Area 2 used for the SANS intensity integration. **c**, **d** The SANS integrated intensities for Area 1 (**a**) and Area 2 (**b**) as a function of magnetic field for *H* || [001] at 5.0 K. The colored lines in (**c**, **d**) are guides to the eye, showing the assignment of each magnetic reflection to either **Q**_1_ or **Q**_1 _+ **Q**_2_. **e**, **f** Magnetic-field dependence of the wavenumber |*Q*| (**e**) and azimuth angle *θ*_*Q*_ (**f**) of the fundamental magnetic modulation vector **Q**_1_. Here, *θ*_*Q*_ is defined as the angle between the **Q**-direction and the [110] axis, as shown in Fig. 2a. **g**, **h** The corresponding data for the higher-order **Q**_1 _+ **Q**_2_ magnetic satellite reflection. The black curves in (**g**, **h**) represent the theoretical |*Q*| and *θ*_*Q*_ values for **Q**_1_ + **Q**_2_ reflection calculated from fundamental magnetic reflections in (**e**, **f**), which agree well with the experimental data. The gray regions between phases I and II indicate the phase coexistence region (see Supplementary Note IV).

Next, in order to identify the magnetic modulation vector in each phase, a series of small-angle neutron scattering (SANS) experiments for the (001) plane at 5.0 K in **H** || [001] of various strengths were performed. Here, both the direction of the incident neutron beam and that of the external magnetic field are parallel to the [001] direction, as shown in Fig. 1d. When the Fourier transform of magnetic structure contains the modulated spin component ($\hat{{{{{{\bf{m}}}}}}}({{{{{\bf{Q}}}}}}){\exp }$[*i***Q∙r**]+c.c.) with $\hat{{{{{{\bf{m}}}}}}}({{{{{\bf{Q}}}}}})$ being a complex vector and c.c. representing complex conjugate, SANS is generally sensitive to the component of $\hat{{{{{{\bf{m}}}}}}}({{{{{\bf{Q}}}}}})$ normal to the magnetic modulation vector **Q**^28^. Figure 2a–d shows the typical SANS patterns obtained in phases I, II, III and IV, respectively. Here, we define *θ*_*Q*_ as the angle between the **Q-**vector and the [110] axis, and **Q**_1_ as the direction of fundamental magnetic modulation vector. In phase I at *H* = 0 (Fig. 2a), we observed magnetic reflections at **Q** = **Q**_1_ along the 〈100〉 directions (i.e. *θ*_*Q*_ ~ 45°). By increasing the magnetic field, the **Q**_1_-direction switches to *θ*_*Q*_ ~ ±5° upon entering phase II (Fig. 2b), and is further aligned along the 〈110〉 direction in phase III (i.e. *θ*_*Q*_ ~ 0°) (Fig. 2c). At higher fields, the **Q**_1_-direction is tilted back to *θ*_*Q*_ ~ ±5° in phase IV (Fig. 2d). Figure 3e, f exhibits the magnetic-field variations of the wavenumber |*Q*| and the azimuth angle *θ*_*Q*_ for the fundamental magnetic reflection **Q** = **Q**_1_, respectively. *θ*_*Q*_ takes distinctive values in each phase, demonstrating the multiple-step reorientation of the fundamental modulation vector in this compound. In phase I, |*Q*| is almost constant at around 0.28 Å^-1^, which corresponds to a modulation period of 2.2 nm. In phases II–IV, |*Q*| is around 0.18 Å^-1^ giving a modulation period of 3.5 nm. Note that the observed fundamental modulation vector **Q**_1_ is always incommensurate, i.e. **Q**_1_ ~ (0, 0.19, 0), (0.073, 0.097, 0), (0.083, 0.083, 0), and (0.070, 0.092, 0) in phases I, II, III, and IV, respectively. Here, reflecting the tetragonal symmetry of the underlying crystal lattice, multiple equivalent magnetic reflections are observed in the SANS pattern for each phase. In phase III, for instance, four fundamental magnetic reflections corresponding to **Q**_1_ = (*q*, *q*, 0), **Q**_2_ = (*q*, -*q*, 0) as well as -**Q**_1_ and -**Q**_2_ are identified (Fig. 2c). Such scattering patterns can originate from a multiple-*Q* spin order hosting multiple number of modulation vectors, or the spatial coexistence of symmetrically equivalent magnetic domains that are related by symmetry elements lost during the phase transition. One straightforward method to distinguish between these two possibilities is the detection of intensity at the **Q**_1_ + **Q**_2_ position, which should appear only in the multiple-*Q* state^17,29,30^. Remarkably, we have identified the clear **Q**_1_ + **Q**_2_ reflection in phases II and III (Fig. 2b, c), demonstrating that phases II and III are double-*Q* states. Figure 3g, h indicates the magnetic-field dependence of the wavenumber |*Q*| and the azimuth angle *θ*_*Q*_ for the **Q**_1_ + **Q**_2_ position. The experimentally observed |*Q*| and *θ*_*Q*_ values for the **Q**_1 _+ **Q**_2_ reflections are consistent with the ones expected from the fundamental reflections (solid lines in Fig. 3g, h). As detailed in Supplementary Notes I, II, and III, similar **Q**_1_ + **Q**_2_ (as well as the associated **Q**_1_ – **Q**_2_) reflections have been identified in the resonant X-ray scattering experiments characterized by better momentum-space resolution. Note that in phase II, the **Q**_1_ and **Q**_2_ directions are not orthogonal to each other, and therefore two double-*Q* states related by the lost four-fold symmetry can coexist as detailed in Supplementary Note II. This explains the observed appearance of eight fundamental reflection spots in phase II (Fig. 2b). By considering a similar magnetic domain formation, the scattering patterns in phases I and IV can be also reasonably explained.

Figure 3c, d exhibits the magnetic-field variations of the SANS integrated intensities measured for Area 1 and Area 2, i.e., the boxed regions along the 〈100〉 and 〈110〉 directions as indicated in Fig. 3a, b, respectively. In phase I with **Q**_1_ || 〈100〉, the SANS intensity is observed only in Area 1. In phases II, III and IV, the SANS intensity emerges in Area 2, reflecting the reorientation of fundamental magnetic modulation vector **Q**_1_. In phases II and III, finite SANS intensity is also observed in Area 1, which corresponds to the **Q**_1 _+ **Q**_2_ reflections originating from the double-*Q* nature of these phases.

**Fig. 4:**
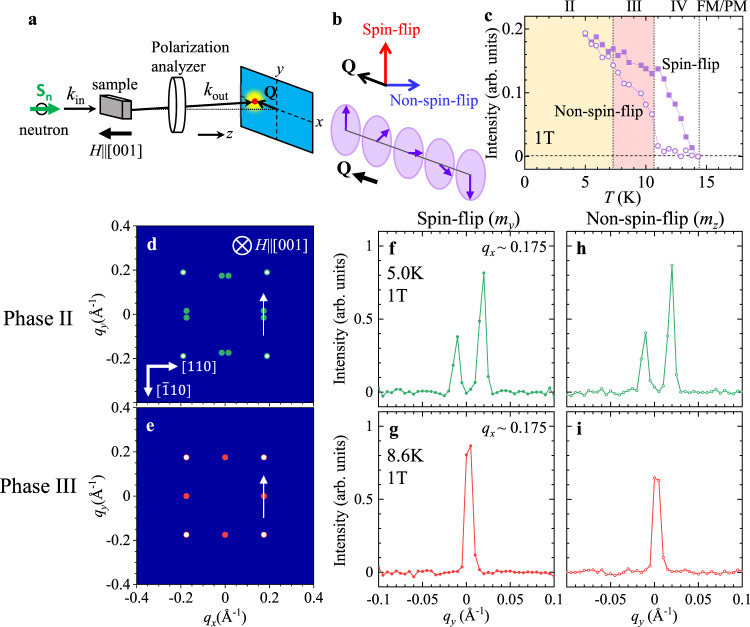
Polarized SANS study of the modulated magnetic states. **a**, **b** Schematic illustration of (**a**) experimental setup and (**b**) the magnetic scattering selection rules. **S**_n_ represents the direction of the neutron polarization, which is aligned parallel or antiparallel with **k**_in_ || [001]. Only a modulated spin component $\hat{{{{{{\bf{m}}}}}}}\left({{{{{\bf{Q}}}}}}\right)$ normal to **Q** can give rise to magnetic neutron scattering. In this setup, spin-flip (SF) scattering arises due to the in-plane component of $\hat{{{{{{\bf{m}}}}}}}\left({{{{{\bf{Q}}}}}}\right)$ perpendicular to both **Q** and **S**_n_ || [001] (red arrow), and non-spin-flip (NSF) scattering arises due to the out-of-plane component of $\hat{{{{{{\bf{m}}}}}}}\left({{{{{\bf{Q}}}}}}\right)$ parallel to **S**_n_ || [001] (blue arrow). **c** Temperature variations of the scattering intensity of SF and NSF channels at 1 T. **d**, **e** Schematic illustration of SANS patterns for phases II and III. The arrows indicate the line-scan directions of (**f**–**i**). **f**–**i** Line-scan profiles for (**f**, **g**) spin-flip and (**h**, **i**) non-spin-flip channels measured in phases II and III at 1 T, which detect in-plane and out-of-plane component of $\hat{{{{{{\bf{m}}}}}}}\left({{{{{\bf{Q}}}}}}\right)$ (i.e. ${\hat{m}}_{y}\left({{{{{\bf{Q}}}}}}\right)$ and ${\hat{m}}_{z}\left({{{{{\bf{Q}}}}}}\right)$), respectively. Here, the scattering intensity is integrated for the range of *q*_*x*_ = 0.175 ± 0.005 Å^-1^. The asymmetry of two peak intensities in phase II (**f**, **h**) is probably due to different populations of the two equivalent magnetic domains.

To investigate the detailed spin orientations in each phase, we have further performed polarized SANS measurements with the experimental setup shown in Fig. 4a. Here, the neutron spin orientation **S**_n_ is aligned parallel or antiparallel to the incident neutron beam (|| [001]), and the intensities of spin-flip and non-spin-flip scattering (*I*_SF_ and *I*_NSF_, respectively) are measured separately (Fig. 4a). In this configuration, *I*_SF_ reflects the component of $\hat{{{{{{\bf{m}}}}}}}({{{{{\bf{Q}}}}}})$ normal to both **S**_n_ || [001] and **Q**, and *I*_NSF_ reflects the component of $\hat{{{{{{\bf{m}}}}}}}({{{{{\bf{Q}}}}}})$ parallel to **S**_n_ || [001] (Fig. 4b)^28^. To access phases II, III and IV, temperature was swept with *μ*_0_*H* = 1 T applied along the [001] direction. In Fig. 4f–i, the line-scan profiles of *I*_SF_ and *I*_NSF_ are plotted for the fundamental magnetic reflections in phases II and III. The corresponding line-scan direction in the reciprocal space is presented in Fig. 4d, e. Magnetic reflections are observed for both *I*_SF_ and *I*_NSF_ channels, which proves that $\hat{{{{{{\bf{m}}}}}}}({{{{{\bf{Q}}}}}})$ possesses both in-plane and out-of-plane components normal to **Q**. This result suggests that $\hat{{{{{{\bf{m}}}}}}}({{{{{\bf{Q}}}}}})$ in phases II and III are characterized by the screw-type spin modulation (Fig. 4b), where their neighboring spins rotate within a plane normal to the magnetic modulation vector **Q**. By considering the double-*Q* nature of phases II and III, their spin textures **m**(**r**)= **s**(**r**)/|**s**(**r**)| should be approximately described as

2 $${{{{{\bf{s}}}}}}({{{{{\bf{r}}}}}})={{{{{{\bf{s}}}}}}}_{0}^{z}+\mathop{\sum}\limits_{i=1,2}\Big({{{{{{\bf{s}}}}}}}_{{{{{{{\bf{Q}}}}}}}_{i}}^{{xy}}{{\cos }}\left({{{{{{\bf{Q}}}}}}}_{i}\cdot {{{{{\bf{r}}}}}}\right)+{{{{{{\bf{s}}}}}}}_{{{{{{{\bf{Q}}}}}}}_{i}}^{z}{{\sin }}\left({{{{{{\bf{Q}}}}}}}_{i}\cdot {{{{{\bf{r}}}}}}\right)\Big)$$

which represents the superposition of screw spin helices characterized by two obliquely or orthogonally arranged magnetic modulation vectors **Q**_1_ and **Q**_2_, respectively. ${{{{{{\bf{s}}}}}}}_{0}^{z}$ is the *H*-induced uniform magnetization component along the [001] axis, and ${{{{{{\bf{s}}}}}}}_{{{{{{{\bf{Q}}}}}}}_{i}}^{{xy}}$ (${{{{{{\bf{s}}}}}}}_{{{{{{{\bf{Q}}}}}}}_{i}}^{z}$) represents the modulated spin component normal to both magnetic modulation vector **Q**_*i*_ and the [001] axis (parallel to the [001] axis). The resultant spin textures for phases II and III are illustrated in Fig. 2f, g, which can be considered as the rhombic and square lattice of magnetic skyrmions, respectively, according to Eq. (1). In Fig. 4c, the temperature dependence of *I*_SF_ and *I*_NSF_ measured at 1 T is summarized. In phase IV above 11 K, the magnetic scattering appears only in the *I*_SF_ channel but not in the *I*_NSF_ channel. It suggests that $\hat{{{{{{\bf{m}}}}}}}({{{{{\bf{Q}}}}}})$ in phase IV consists only of the in-plane component normal to **Q**. A similar conclusion is also obtained from the resonant elastic X-ray scattering results for phase IV, as discussed in Supplementary Note I. Note that additional neutron scattering data in Supplementary Fig. 8 indicate the existence of very weak but discernible **Q**_1_ + **Q**_2_ reflection in phase IV, which suggests the formation of double-*Q* vortex-lattice spin state described by the superposition of two sinusoidally-modulated in-plane spin components (Fig. 2h). For the detailed discussion on the spin texture for phase IV, see Supplementary Note VII and VIII.

## Discussion

In the following, we discuss the microscopic origin for the appearance of square and rhombic SkL states in EuAl_4_. Since the crystal structure of EuAl_4_ is centrosymmetric, the contribution of DM interaction should be absent and some different mechanism must be considered. According to the recent theoretical studies^19–22^, the SkL formation in centrosymmetric rare-earth compounds are approximately explained by the combination of Ruderman-Kiettel-Kasuya-Yosida (RKKY) interaction $\hat{{{{J}}}}\left({{{{{\bf{Q}}}}}}\right)[\hat{{{{{{\bf{m}}}}}}}\left({{{{{\bf{Q}}}}}}\right)\cdot \hat{{{{{{\bf{m}}}}}}}\left({{{{{\boldsymbol{-}}}}}}{{{{{\bf{Q}}}}}}\right)]$ and four-spin interaction $\hat{K}\left({{{{{\bf{Q}}}}}}\right){[\hat{{{{{{\bf{m}}}}}}}\left({{{{{\bf{Q}}}}}}\right)\cdot \hat{{{{{{\bf{m}}}}}}}(-{{{{{\bf{Q}}}}}})]}^{2}$, which are derived from the Kondo lattice model consisting of localized magnetic moments and itinerant electrons. Here, the former RKKY term stabilizes the magnetic modulation with a specific wave vector, and the latter four-spin interaction term enhances the stability of the multiple-*Q* orders. This model well explains the recently reported square SkL formation in a centrosymmetric tetragonal magnet GdRu_2_Si_2_^17,23^, and similar mechanism would be also relevant for EuAl_4_ having the same tetragonal crystal structure.

Compared to the known skyrmion-hosting materials, the present EuAl_4_ is characterized by several unique features. First, EuAl_4_ shows multiple-step reorientation of the fundamental magnetic modulation vector as a function of external magnetic field, which leads to the appearance of not only square, but also rhombic form of SkL states. These behaviors are distinctive from the previously reported centrosymmetric Gd-compounds^16–18^, where the magnetic modulation vector is always fixed along the one specific direction and the associated SkL reflects the symmetry of underlying crystal lattice. (For example, only the square SkL appears for tetragonal GdRu_2_Si_2_ with **Q** ||〈100〉^17^). In general, the orientation and length of stable magnetic modulation vector are determined by the peak position in $\hat{J}\left({{{{{\bf{Q}}}}}}\right)$ reflecting the character of the associated Fermi surfaces or electronic structure. The presently observed multiple-step **Q**-reorientation in EuAl_4_ implies that almost comparable amplitude of $\hat{J}\left({{{{{\bf{Q}}}}}}\right)$ peaks exist for several **Q**-positions. In Supplementary Note IX and Supplementary Fig. 10, we performed theoretical simulation based on this assumption, which turned out to well reproduce the observed *H*-induced transition between rhombic and square SkL states. This suggests that the symmetry of SkL sensitively depends on the electronic structure^20^ and potentially can be controlled by chemical substitution or external stimuli, which would be a unique feature of itinerant-electron-mediated mechanism compared with the traditional DM-based mechanism.

Second, EuAl_4_ is the first example of centrosymmetric binary compound to host skyrmion spin texture, which just consists of the alternate stacking of magnetic Eu layer and nonmagnetic Al layer (Fig. 1a). The interactions among the Eu^2+^ localized magnetic moments are mediated by itinerant electrons, whose Fermi surfaces are mostly governed by Al according to the recent ARPES (angle-resolved photoemission spectroscopy) measurements^31^. The above features highlight EuAl_4_ as the simple model system to embody the unique skyrmion formation mechanisms mediated by itinerant electrons. It is remarkable that even such a minimum centrosymmetric binary compound can host a variety of distinctive skyrmion orders with intricate multiple-step **Q**-reorientation. The present experimental results demonstrate that rare-earth intermetallics with delicate balance of magnetic interactions^19–22,32–36^ can be a promising platform to realize/control the competition of multiple topological magnetic phases in a single compound, which would be a good guideline for further search of exotic materials with emergent functional responses.

## Methods

### Sample preparation and characterization

Single crystals of EuAl_4_ were grown by the Al self-flux method. The samples were characterized by powder X-ray diffraction, which confirmed the purity of the grown crystals. Crystal orientations were determined by Laue X-ray diffraction, and then the samples were cut out from the crystal with a wire saw and carefully mechanically polished.

### Magnetization and resistivity measurements

Magnetization was measured using a SQUID magnetometer (Magnetic Property Measurement System, Quantum Design). The longitudinal (*ρ*_*xx*_) and Hall (*ρ*_*yx*_) resistivities were measured using the ac-transport option in a physical property measurement system (PPMS, Quantum Design). Measurements of magnetic and electrical transport properties were performed on the same crystal with a dimension of 1 mm by 0.9 mm by 0.5 mm.

### SANS measurements

SANS measurements were carried out at the time-of-flight type small-and-wide-angle neutron scattering instrument TAIKAN (BL15) at Materials and Life Science Experimental Facility (MLF) in Japan Proton Accelerator Research Complex (J-PARC)^37^. A thin crystal with a thickness of 0.2 mm having the widest surface of 5 mm by 2.5 mm parallel to the (001) plane was installed in a cryostat equipped with a horizontal-field superconducting magnet. An incident neutron beam with a wavelength ranging from 0.7 to 7.7 Å was exposed on the sample. The incident beam was always directed along the [001] axis within the accuracy of 2 degrees. The typical measuring time for a SANS pattern was 200 s. The SANS patterns (Fig. 2) were measured with rotating and tilting the sample up to 2 degrees. Background data were obtained at 15.5 K above *T*_*c*_ in a magnetic field of 0.05 T, and subtracted from the data obtained at low *T* to leave only the magnetic signal. As for the magnetic-field scans, SANS signals were collected with continuously increasing magnetic field at a sweeping rate of 0.01 T/min.

In the SANS experiments with longitudinal polarization analysis, a polarized incident neutron beam was obtained by a supermirror polarizer. The neutron spin polarization at the sample position was controlled to be parallel to the [001] axis of the sample by guide fields and the horizontal-field superconducting magnet, which applied a magnetic field of 1.0 T. A supermirror spin analyzer was set between the sample and the detectors, and was used to separate the spin-flip (SF) and non-spin-flip (NSF) scattering signals. Total beam polarization measured using a direct beam was 0.85 for the neutrons with the wavelengths longer than 3.5 Å. The incident and scattering angles for the fundamental magnetic Bragg reflections near the (*H,H*,0) line were tuned so that the wavelengths for the Bragg reflections were approximately 3.5 Å. The mixing of the SF and NSF signals was corrected taking into account of the total beam polarization.

### X-ray measurements

Resonant elastic X-ray scattering experiment was carried out using circularly polarized X-rays of an incoming photon energy tuned approximately to the Eu *L*_2_ absorption edge (7617 eV) in Laue-transmission geometry on beamline P09 at the PETRA-III synchrotron, DESY. A (001)-oriented thin plate of EuAl_4_ with a thickness of 10 μm and an area of 600 μm by 300 μm was attached to a Si_3_N_4_ substrate (almost X-ray transparent). The substrate was attached to a sample holder mounted at the bottom of a long probe and inserted into the variable temperature insert (VTI). The X-ray beam was focused to a spot size of ~70 × 200 μm^2^. Diffraction patterns were recorded by a two-dimensional detector PILATUS300K by rocking the sample. A magnetic field was applied parallel to the incident X-ray beam and perpendicular to the thin plate (|| [001]) by a cryogen-free vector magnet. Background data were measured at 14.5 K where no magnetic scattering was observed.

## Supplementary information

Supplementary Infromation

## Data Availability

The data presented in this study are available from the corresponding author upon reasonable request.
