# Supplementary material for: Square and rhombic lattices of magnetic skyrmions in a centrosymmetric binary compound
Source: Nat Commun. 2022 Mar 30;13:1472. doi: 10.1038/s41467-022-29131-9 (PMC8967868; doi:10.1038/s41467-022-29131-9)
Supplement: Supplementary file 1 — Supplementary Infromation [file 41467_2022_29131_MOESM1_ESM.pdf]

Supplementary Information:
Square and rhombic lattices of magnetic skyrmions in a centrosymmetric binary compound

Rina Takagi[1,2,3,4*], Naofumi Matsuyama[1], Victor Ukleev[5], Le Yu[5,6,7], Jonathan S. White[5], Sonia Francoual[8], José R. L. Mardegan[8], Satoru Hayami[1,3], Hiraku Saito[9], Koji Kaneko[10,11], Kazuki Ohishi[12], Yoshichika Ōnuki[4], Taka-hisa Arima[4,13], Yoshinori Tokura[1,4,14], Taro Nakajima[4,9], Shinichiro Seki[1,2,3,4]

[1] *Department of Applied Physics, University of Tokyo, Tokyo 113-8656, Japan,*

[2] *Institute of Engineering Innovation, University of Tokyo, Tokyo 113-0032, Japan,*

[3] *PRESTO, Japan Science and Technology Agency (JST), Kawaguchi 332-0012, Japan,*

[4] *RIKEN Center for Emergent Matter Science (CEMS), Wako 351-0198, Japan,*

[5] *Laboratory for Neutron Scattering and Imaging (LNS), Paul Scherrer Institute (PSI), CH-5232 Villigen, Switzerland,*

[6] *Laboratory for Ultrafast Microscopy and Electron Scattering (LUMES), Institute of Physics, École Polytechnique Fédérale de Lausanne (EPFL), CH-1015 Lausanne, Switzerland,*

[7] *Laboratory of Nanoscale Magnetic Materials and Magnonics (LMGN), Institute of Materials, École Polytechnique Fédérale de Lausanne (EPFL), CH-1015 Lausanne, Switzerland,*

[8] *Deutsches Elektronen-Synchrotron DESY, Notkestraße 85, 22607 Hamburg, Germany,*

[9] *The Institute for Solid State Physics, University of Tokyo, Kashiwa 277-8561, Japan,*

[10] *Materials Sciences Research Center, Japan Atomic Energy Agency, Tokai 319-1195, Japan,*

[11] *J-PARC Center, Japan Atomic Energy Agency, Tokai 319-1195, Japan,*

[12] *Neutron Science and Technology Center, Comprehensive Research Organization for Science and Society (CROSS), Tokai 319-1106, Japan,*

[13] *Department of Advanced Materials Science, University of Tokyo, Kashiwa 277-8561, Japan,*

[14] *Tokyo College, University of Tokyo, Tokyo 113-8656, Japan.*

I. Resonant X-ray scattering experiments for $EuAl_4$

In this section, we present the results of resonant elastic X-ray scattering experiments, performed for the (001) plane at 5.5 K under various amplitudes of $\mathbf{H}$ || [001]. Here, the directions of incident X-ray beam and external magnetic field are fixed orthogonal to the sample surface (i.e. parallel to the [001] direction) as shown in Supplementary Fig. 1a. When the Fourier transform of magnetic structure contains the modulated spin component $(\hat{\mathbf{m}}(\mathbf{Q}) \exp[i\mathbf{Q} \cdot \mathbf{r}] + \text{c.c.})$ with $\hat{\mathbf{m}}(\mathbf{Q})$ being a complex vector and c.c. representing complex conjugate, the corresponding magnetic X-ray scattering intensity $I(\mathbf{Q})$ can be described as $I(\mathbf{Q}) \propto |(\mathbf{e}_i \times \mathbf{e}_f) \cdot \hat{\mathbf{m}}(\mathbf{Q})|^2$, where $\mathbf{e}_i$ and $\mathbf{e}_f$ represent the polarization vectors of incident and scattered beams, respectively [S1]. Since the scattered beam is approximately parallel to the incident beam in the small-angle scattering geometry, the component of $\hat{\mathbf{m}}(\mathbf{Q})$ parallel to the out-of-plane [001] direction is mainly detected in the present measurements. Supplementary Fig. 1b-e shows the typical X-ray scattering patterns in phases I, II, III and IV, where the white square outlines the area experimentally measured in our setup. Based on these data, the magnetic-field variations of the scattering intensity, the wavenumber $|Q|$ and the azimuth angle θ_Q for the fundamental magnetic reflection $\mathbf{Q} = \mathbf{Q}_1$ are summarized in Supplementary Fig. 2a-c. Here, we defined θ_Q as the angle between $\mathbf{Q}$ and the [110] axis. In the process of H-induced successive magnetic phase transitions I→II→III, the multiple-step reorientation of $\mathbf{Q}$-vector is identified, and their $|Q|$ and θ_Q values agree well with the ones observed by the neutron scattering experiments (Fig. 3e,f in the main text). Note that phase IV doesn't show any magnetic reflection in the X-ray scattering profiles (Supplementary Fig. 1e), in contrast with the SANS results showing eight fundamental magnetic reflection spots (Fig. 2d in the main text). Since the present X-ray (neutron) measurements can detect only the out-of-plane (both in-plane and out-of-plane) component of $\hat{\mathbf{m}}(\mathbf{Q})$, the present results suggest that phase IV possesses only the in-plane component of $\hat{\mathbf{m}}(\mathbf{Q})$. In general, the SANS intensity reflects the component of $\hat{\mathbf{m}}(\mathbf{Q})$ normal to $\mathbf{Q}$, and therefore each fundamental modulation vector $\mathbf{Q}$ is characterized by the sinusoidally modulated spin component normal to both $\mathbf{Q}$ and the [001] axis in phase IV (The detailed spin texture in phase IV is discussed in Supplementary Note VII, VIII and IX). For phase I, the appearance of magnetic reflections in the X-ray scattering results suggests the existence of out-of-plane component of $\hat{\mathbf{m}}(\mathbf{Q})$. This is consistent with the polarized neutron scattering results in Supplementary Note VIII and Supplementary Fig. 9, which identified phase I as the screw spin state as shown in Fig. 2e in

the main text.

II. Magnetic domains in phases I, II, and IV

In this section, we discuss the magnetic domains in phases I, II, and IV. When the spin texture breaks the symmetry of the original crystal lattice, multiple energetically-degenerated magnetic domains appear. These domains are related to each other by the symmetry elements that are lost during the phase transition process. For example, as discussed in the main text, phase II is the double-Q state satisfying two distinctive fundamental magnetic modulation vectors making an angle of 80° with respect to each other. Its reciprocal-space scattering pattern and corresponding real-space spin texture calculated based on Eq. (2) in the main text are indicated in Supplementary Fig. 3a,d, where the appearance of four fundamental magnetic reflection spots are expected from a single domain (domain 1). Since this spin texture breaks the four-fold symmetry of the original $EuAl_4$ crystal lattice, there will appear another equivalent magnetic domain (i.e. domain 2) that can be obtained by the 90° rotation of domain 1 (Supplementary Fig. 3b,e). By assuming the equal population of these two domains, the appearance of eight fundamental magnetic reflection spots as shown in Supplementary Fig. 3c is expected. This scenario well explains the observed resonant X-ray scattering and SANS patterns in phase II (Supplementary Fig. 1c and Fig. 2b in the main text). The above argument about the magnetic domain formation can be applied to phases I and IV, in the latter of which broken four-fold and mirror symmetry explains the appearance of eight magnetic reflection spots as seen in Fig. 2d in the main text.

III. Analysis of $\mathbf{Q}_1+\mathbf{Q}_2$ and $\mathbf{Q}_1-\mathbf{Q}_2$ magnetic reflections in phases II and III

In this section, we discuss the $\mathbf{Q}_1+\mathbf{Q}_2$ and $\mathbf{Q}_1-\mathbf{Q}_2$ magnetic reflections in the resonant X-ray scattering profiles. As discussed in the main text, phases II and III are the double-$\mathbf{Q}$ states and host the higher-order $\mathbf{Q}_1+\mathbf{Q}_2$ reflections in their SANS profiles (Fig. 2b,c in the main text). Similar $\mathbf{Q}_1+\mathbf{Q}_2$ reflections are also observed in the corresponding X-ray scattering profiles (Supplementary Fig. 1c,d). Supplementary Fig. 2d-f indicates the magnetic field dependence of the X-ray scattering intensity, the wavenumber $|Q|$, and the azimuth angle θ_Q for the $\mathbf{Q}_1+\mathbf{Q}_2$ reflection. The $\mathbf{Q}_1+\mathbf{Q}_2$ reflections are present in phases II and III but absent in phases I. These

features confirm that the observed higher-order reflections originate from the double-Q nature of phases II and III, and not the double-scattering process. Note that the experimentally observed $|Q|$ and θ_Q values for $\mathbf{Q}_1+\mathbf{Q}_2$ reflections are consistent with the ones theoretically calculated from the fundamental reflections (solid lines in Supplementary Fig. 2e,f).

In Supplementary Fig. 4, schematic illustration of reciprocal-space scattering pattern and the line-scan profiles of the resonant X-ray and neutron scattering measured along the [100] direction are plotted for each phase. In phase II (Supplementary Fig. 4b,e,h), two magnetic reflection peaks are identified in the line-scan profiles, where the ones with smaller and larger $|Q|$ values represent the $\mathbf{Q}_1-\mathbf{Q}_2$ reflection from domain 1 (Supplementary Fig. 3a) and the $\mathbf{Q}_1+\mathbf{Q}_2$ reflection from domain 2 (Supplementary Fig. 3b), respectively. They merge into a single $\mathbf{Q}_1+\mathbf{Q}_2$ peak in phase III, where the $\mathbf{Q}_1$ and $\mathbf{Q}_2$ vectors become exactly orthogonal with respect to each other (Supplementary Fig. 4c,f,i). Note that the peak in phase I represents the fundamental magnetic reflection along the [100] direction, i.e., not the higher-order $\mathbf{Q}_1+\mathbf{Q}_2$ reflection.

IV. Phase boundary between phases I and II

Supplementary Fig. 5a,b shows the magnetic-field variations of the SANS integrated intensities, taken for the boxed regions, Area 1 and Area 2 shown in Supplementary Fig. 5c,d, respectively. In phase I, the intensity is finite only in Area 1, reflecting the fundamental magnetic reflection $\mathbf{Q} = \mathbf{Q}_1$ along the $\langle 100 \rangle$ reciprocal lattice direction. In the intermediate fields between phases I and II indicated by the gray regions in Supplementary Fig. 5a,b (corresponding to those in Fig. 3 in the main text), the intensity of Area 2 starts to increase, reflecting the transition into phase II, while that of Area 1 gradually decreases. Such anticorrelation of intensity between phases I and II implies that phase I is gradually replaced by phase II. During this phase transition process, the intensity of Area 1 should contain the contributions not only from the $\mathbf{Q}_1+\mathbf{Q}_2$ reflection of phase II but also from the $\mathbf{Q}_1$ reflection of phase I. Here, the phases I and II are characterized by the screw spin texture (Supplementary Fig. 5e) and the rhombic skyrmion lattice (Supplementary Fig. 5g), respectively, and the spatial coexistence of these two phases is expected in the intermediate state.

In the main text, we assumed that the spin texture $\mathbf{m}(\mathbf{r})=\mathbf{s}(\mathbf{r})/|\mathbf{s}(\mathbf{r})|$ of phase II can be approximately described as

$$\mathbf{s}(\mathbf{r}) = \mathbf{s}_0^z + \sum_{i=1,2} (\mathbf{s}_{\mathbf{Q}_i}^{xy} \cos(\mathbf{Q_i} \cdot \mathbf{r}) + \mathbf{s}_{\mathbf{Q}_i}^{z} \sin(\mathbf{Q_i} \cdot \mathbf{r})), \qquad \text{(S1)}$$

which represents the superposition of screw spin helices characterized by two obliquely arranged magnetic modulation vectors $\mathbf{Q}_1$ and $\mathbf{Q}_2$, respectively (Supplementary Fig. 5g). $\mathbf{s}_0^z$ is the H-induced uniform magnetization component along the [001] axis, and $\mathbf{s}_{\mathbf{Q}_i}^{xy}$ ($\mathbf{s}_{\mathbf{Q}_i}^{z}$) represents the modulated spin component normal to both magnetic modulation vector $\mathbf{Q}_i$ and the [001] axis (parallel to the [001] axis). For $EuAl_4$, the amplitude of $\mathbf{m}(\mathbf{r})$ should be constant due to the localized character of magnetic moments on Eu^{2+} sites, and the associated normalization process leads to the appearance of higher-order $\widehat{\mathbf{m}}(\mathbf{Q}_1 + \mathbf{Q}_2)$ and $\widehat{\mathbf{m}}(\mathbf{Q}_1 - \mathbf{Q}_2)$ components. Note that the experimentally observed scattering intensity for the $\mathbf{Q}_1$+$\mathbf{Q}_2$ reflection is considerably larger than the $\mathbf{Q}_1$–$\mathbf{Q}_2$ reflection in phase II (Supplementary Fig. 4e,h), which implies that the introduction of $\widehat{\mathbf{m}}(\mathbf{Q}_1 + \mathbf{Q}_2)$ component may be necessary. In this case, Eq. (S1) is replaced by

$$\mathbf{s}(\mathbf{r}) = \mathbf{s}_0^z + \sum_{i=1,2} (\mathbf{s}_{\mathbf{Q}_i}^{xy} \cos(\mathbf{Q}_i \cdot \mathbf{r}) + \mathbf{s}_{\mathbf{Q}_i}^{z} \sin(\mathbf{Q}_i \cdot \mathbf{r}))$$
$$+ \mathbf{s}_{\mathbf{Q}_1+\mathbf{Q}_2}^{xy} \cos((\mathbf{Q}_1 + \mathbf{Q}_2) \cdot \mathbf{r}) + \mathbf{s}_{\mathbf{Q}_1+\mathbf{Q}_2}^{z} \sin((\mathbf{Q}_1 + \mathbf{Q}_2) \cdot \mathbf{r}), \qquad \text{(S2)}$$

which represents the superposition of the screw spin helices characterized by magnetic modulation vectors $\mathbf{Q}_1$, $\mathbf{Q}_2$, and $\mathbf{Q}_1$+$\mathbf{Q}_2$. In general, skyrmion lattices are created by pinching off the helical magnetic stripes. To follow this picture, we have chosen the relative phase so that the skyrmion core position defined by $\mathbf{Q}_1$ and $\mathbf{Q}_2$ locates at the middle of negative m_z (antiparallel to external magnetic field) region of $\mathbf{Q}_1$+$\mathbf{Q}_2$ screw spin modulation. The resultant spin texture is shown in Supplementary Fig. 5f, where elliptically deformed skyrmions are further elongated along the direction perpendicular to $\mathbf{Q}_1$+$\mathbf{Q}_2$ compared with the one based on Eq. (S1) (Supplementary Fig. 5g). In either case, the phase II represents the rhombic skyrmion lattice state. The detailed spin texture for phase II and the associated magnetic phase transition process are issues for the future study.

V. Analysis of Hall resistivity profile

In this section, we discuss the magnetic field dependence of Hall resistivity in Fig. 1g in the main text. In general, the Hall resistivity $\rho_{yx} = \rho_{yx}^{\mathrm{N}} + \rho_{yx}^{\mathrm{A}} + \rho_{yx}^{\mathrm{T}}$ consists of normal Hall term $\rho_{yx}^{\mathrm{N}} = R_0 \mu_0 H$ proportional to H, anomalous Hall term $\rho_{yx}^{\mathrm{A}} = R_S M$ proportional to M, and topological Hall term $\rho_{yx}^{\mathrm{T}} = P R_0 B_{\mathrm{eff}}$ proportional to the emergent magnetic field B_{eff}. R_0

and R_s are normal and anomalous Hall coefficients, respectively, and P is the spin-polarization ratio of the conduction electron [S2].

When the rigid band structure is assumed, the anomalous Hall term originating from the intrinsic and skew scattering mechanism is generally described as $\rho_{yx}^{\mathrm{A}} \propto \rho_{xx}^{2}M$ and $\rho_{yx}^{\mathrm{A}} \propto \rho_{xx}M$, respectively [S3]. In Supplementary Fig. 6c, H-dependence of ρ_{yx}^{A} estimated from these formula and experimental M and ρ_{xx} profiles at 4 K are plotted, which shows considerable deviation from the experimental ρ_{yx} profile (Supplementary Fig. 6b). This argument is also applied to the Hall conductivity, σ_{xy} , calculated as $\sigma_{xy} = \rho_{yx}/\left({\rho_{xx}}^{2} + {\rho_{yx}}^{2}\right)$ (Supplementary Fig. 6a).

At this stage, it is not straightforward to conclude the detailed origin of the observed discrepancy. This is because the electronic structure (and associated carrier density and Fermi-surface properties) in rare-earth compounds sensitively depends on the magnetic structure [S4], and therefore the normal and anomalous Hall term ρ_{yx}^{N} and ρ_{yx}^{A} should be characterized by more complicated behavior than the above formula. Moreover, recent theory has proposed that the reciprocal-space and real-space Berry phases are closely linked, which gives rise to the so-called chiral Hall effect derived from noncollinear magnetism [S5]. In the present compound, this chiral Hall effect may also provide a sizable contribution to the Hall resistivity.

In principle, the appearance of topological Hall term ρ_{yx}^{T} can be expected in the skyrmion lattice phases, i.e. phases II and III. Here, the emergent magnetic field $B_{\mathrm{eff}} = \Phi_0\Phi$ originates from the quantum-mechanical Berry phase gained by the conduction electrons passing through skyrmion spin textures, and Φ and $\Phi_0 = h/e$ represent skyrmion density and flux quantum, respectively [S2]. If we tentatively assume the typical value of spin polarization ratio $P \sim 0.01$ previously estimated for the other Gd-based centrosymmetric skyrmion-hosting materials [S6,S7], the expected amplitude of topological Hall term ρ_{yx}^{T} for phases II and III with the modulation period of 3.5 nm would be in order of $\left|\Delta\rho_{yx}^{\mathrm{T}}\right| \sim 0.16\,\mu\Omega\mathrm{cm}$. For the full understanding of the experimental ρ_{yx} profile and the quantitative evaluation of topological Hall term in $EuAl_4$, however, further theoretical analysis and the detailed information on the electronic structure would be necessary.

VI. SANS patterns for each magnetic phase at 5 K

The SANS data were collected by rotating and tilting the sample such that the magnetic

diffraction signal is rotated through the Ewald sphere. The typical SANS patterns at 5 K in Fig. 2a-d in the main text were obtained by combining four distinctive images measured at each rotating (ω) and tilting (χ) angles as shown in Supplementary Fig. 7.

VII. SANS patterns and spin textures for phases IV

In this section, we discuss the detail of the SANS pattern and corresponding spin texture for the temperature (T) – magnetic field (H) region labeled as phase IV. Supplementary Fig. 8 shows the SANS patterns for various T and H values in phase IV. At 5.0 K and 1.4 T (Supplementary Fig. 8b), we observed two distinctive fundamental magnetic modulation vectors $\mathbf{Q}_1$ and $\mathbf{Q}_2$ making an angle of 80° with respect to each other (corresponding to Fig. 2d in the main text). Here, very weak but clear $\mathbf{Q}_1+\mathbf{Q}_2$ and $\mathbf{Q}_1-\mathbf{Q}_2$ magnetic reflections can also be identified on the log scale (Supplementary Fig. 8c), indicating that phase IV is the double-Q state. As discussed in Supplementary Note I, this state is characterized by only the in-plane component of $\widehat{\mathbf{m}}(\mathbf{Q})$, and can be considered as the superposition of two orthogonally modulated sinusoidal spin component described by

$$\mathbf{s}(\mathbf{r}) = \mathbf{s}_0^z + \sum_{i=1,2} (\mathbf{s}_{\mathbf{Q}_i}^{xy} \sin(\mathbf{Q}_i \cdot \mathbf{r})). (S3)$$

It represents the rhombic form of vortex lattice as shown in Supplementary Fig. 8g. At higher-T region, the fundamental modulation vectors become orthogonal to each other and four-fold symmetry is recovered (Supplementary Fig. 8d,e,f). For the latter state, the polarized neutron scattering results in Fig. 4c in the main text confirms that $\widehat{\mathbf{m}}(\mathbf{Q})$ contains only in-plane spin component. Therefore, its spin texture is also described by Eq. (S3), representing the square vortex lattice state as shown in Supplementary Fig. 8h. As discussed in Supplementary Note IX and Supplementary Fig. 10, our additional theoretical simulation suggests that the above two vortex-lattice spin states with and without four-fold symmetry are energetically almost degenerated. Since we couldn't identify their clear phase boundary from magnetization and electrical transport measurements, we tentatively assigned both of them as phase IV. Some additional discussion on phase IV is also provided in Supplementary Notes VIII and IX.

VIII. Polarized neutron scattering for phases I, IV, V, and VI

To elaborate on the magnetic structures in phases I, IV, V, and VI, additional neutron

diffraction measurements with longitudinal polarization analysis were performed at zero field with a triple-axis spectrometer PONTA at JRR-3, Japan. A polarized incident neutron beam with an energy of 13.7 meV was obtained by a Heusler monochromator. The incident neutron spin polarization $\mathbf{S}_n$ was controlled by a spin flipper and a Helmholtz coil. The flipping ratio of the polarized neutron beam was 14~17. The neutron scatterings with the spin flipper on and off correspond to the spin-flip (SF) and non-spin-flip (NSF) scattering, respectively, where a Heusler analyzer was used to separate the SF and NSF scattering signals.

The experiments were carried out in two different scattering planes, specifically $(h,0,l)$ and (h,h,l) planes, in which we measured line scan profiles of the magnetic Bragg reflections at (q, 0, 0) and (q, q, 0) along the (h,0,0) and (h,h,0) directions, respectively. These setups are schematically drawn in Supplementary Fig. 9a,b. The neutron spin polarization vector $\mathbf{S}_n$ was set to be perpendicular to the scattering plane in both setups. Therefore, in the former setup, the intensities measured in the SF and NSF scattering channels correspond to the Fourier-transformed spin components $\hat{\mathbf{m}}(\mathbf{Q})$ along the $(\mathbf{S}_\mathrm{n} \times \mathbf{Q}) \parallel [001]$ and $\mathbf{S}_\mathrm{n} \parallel [010]$ directions, respectively. Similarly, in the latter setup, the SF and NSF intensities correspond to the Fourier-transformed spin components $\hat{\mathbf{m}}(\mathbf{Q})$ along the $(\mathbf{S}_\mathrm{n} \times \mathbf{Q}) \parallel [001]$ and $\mathbf{S}_\mathrm{n} \parallel [\bar{1}10]$ directions, respectively.

The temperature dependences of the q value and the integrated intensity for SF and NSF scatterings for the (q, 0, 0) and (q, q, 0) peaks are summarized in Supplementary Fig. 9c-h. In phases I and V, we observed fundamental magnetic reflections $\mathbf{Q}_1$ = (q, 0, 0), and the q value is jumped on the transition between the two phases. For both phases I and V, the intensity of the SF scattering is almost the same as that of the NSF scattering, indicating that $\hat{\mathbf{m}}(\mathbf{Q_1})$ possesses both [001] and [010] components normal to $\mathbf{Q}_1$. In phases VI and IV, fundamental magnetic peaks appear at $\mathbf{Q}_1$ = (q, q, 0), reflecting the reorientation of fundamental magnetic modulation vector. This is in good agreement with the result in Ref. [S8]. The (q, q, 0) magnetic scattering appears only in the NSF channel but not in the SF channel, indicating that $\hat{\mathbf{m}}(\mathbf{Q_1})$ possesses $[\bar{1}10]$ components normal to $\mathbf{Q}_1$ but don't have the [001] component. In phase VI, we also identified (q, 0, 0) peak representing the $\mathbf{Q}_1$+$\mathbf{Q}_2$ reflection. This proves that phase VI is the double-Q state. Since the $\mathbf{Q}_1$+$\mathbf{Q}_2$ peak appears only in the SF channel but not in the NSF one, it is suggested that $\hat{\mathbf{m}}(\mathbf{Q_1} + \mathbf{Q_2})$ contains the [001] component, but don't have the [010] component in phase VI.

On the basis of the above results, schematic SANS patterns for each phase are illustrated

in Supplementary Fig. 9i-l. Since we didn't observe the $\mathbf{Q}_1$+$\mathbf{Q}_2$ reflections, phases I and V are the single-Q state described by

$$\mathbf{s}(\mathbf{r}) = \mathbf{s}_{\mathbf{Q}_1}^{xy} \cos(\mathbf{Q_1} \cdot \mathbf{r}) + \mathbf{s}_{\mathbf{Q}_1}^{z} \sin(\mathbf{Q_1} \cdot \mathbf{r}), \qquad \text{(S4)}$$

corresponding to the screw spin texture with $\boldsymbol{Q}_1 = (q, 0, 0)$ (Supplementary Fig. 9i,j). Phase VI is identified as the double-Q state and can be described by

$$\mathbf{s}(\mathbf{r}) = \sum_{i=1,2} (\mathbf{s}_{\mathbf{Q}_i}^{xy} \sin(\mathbf{Q}_i \cdot \mathbf{r}) + \mathbf{s}_{\mathbf{Q}_1+\mathbf{Q}_2}^{z} \sin((\mathbf{Q_1} + \mathbf{Q_2}) \cdot \mathbf{r}) + \mathbf{s}_{\mathbf{Q}_1-\mathbf{Q}_2}^{z} \sin((\mathbf{Q_1} - \mathbf{Q_2}) \cdot \mathbf{r})), \qquad \text{(S5)}$$

which represents the superposition of four sinusoidal spin orders characterized by magnetic modulation vectors $\mathbf{Q}_1 = (q, q, 0)$, $\mathbf{Q}_2 = (q, -q, 0)$, $\mathbf{Q}_1 + \mathbf{Q}_2$, and $\mathbf{Q}_1 - \mathbf{Q}_2$ (Note that the relative phase of each modulating component is arbitrarily chosen since it cannot be determined from diffraction experiments). The spin texture based on Eq. (S5) is shown in Supplementary Fig. 9k, which turned out to be identical with the one proposed in a recent theoretical work based on the Heisenberg model on a square lattice with competing interactions (MX-II state in Ref. [S9]). As discussed in Supplementary Note VII, phase IV can be also considered as the double-Q state. Its spin texture at $H = 0$ can be described by

$$\mathbf{s}(\mathbf{r}) = \sum_{i=1,2} (\mathbf{s}_{\mathbf{Q}_i}^{xy} \sin(\mathbf{Q}_i \cdot \mathbf{r})), \text{(S6)}$$

representing the superposition of two sinusoidally modulated spin components with $\mathbf{Q}_1 = (q, q, 0)$ and $\mathbf{Q}_2 = (q, -q, 0)$. The resultant spin texture is shown in Supplementary Fig. 9l, indicating the square vortex-lattice spin state. Here, tiny orthorhombic distortion of crystal structure (phases I & V) from the high-temperature tetragonal structure (phases VI & IV) has been reported in Ref. [S10]. Considering the anisotropy of the spin textures in Supplementary Fig. 9i-l, the reported orthorhombic structural distortion probably reflects the symmetry change of the spin texture.

IX. Theoretical simulation of spin configuration

To investigate the microscopic origins of the experimentally observed square and rhombic skyrmion lattice (SkL), we performed simulated annealing for the two-dimensional square lattice system based on the effective spin Hamiltonian derived from the Kondo lattice model [S11], which is given by

$$\mathcal{H} = 2\sum_{\nu}\left(-\hat{J}\lambda_{\nu} + \frac{\hat{K}}{N}\lambda_{\nu}^{2}\right) - H\sum_{i} m_{p}^{z}, \text{ (S7)}$$

where $\lambda_{\nu} = \sum_{i,j}\Gamma_{Q_{\nu}}^{ij}\hat{m}_{i}(\mathbf{Q}_{\nu})\hat{m}_{j}(\mathbf{Q}_{-\nu})\ (i,j = [100],[010],[001])$. $\hat{\mathbf{m}}(\mathbf{Q}_{\nu})$ is the Fourier transformation of the classical localized spin m_p at the position p ($|m_p| = 1$), and N is the system size. The first term represents the bilinear (RKKY) exchange interaction $\hat{J}$ and the second term represents the biquadratic exchange interaction $\hat{K}$ defined in momentum space. The wave vector $\mathbf{Q}_{\nu}$ are set by the nesting of the Fermi surfaces. The magnetic phases in Supplementary Fig. 10 are calculated under the condition that the bare susceptibility χ_q shows the first maxima at $\mathbf{Q}_{\alpha1} = (2\pi/5, 0)$ and $\mathbf{Q}_{\alpha2} = (0, 2\pi/5)$ and the second maxima at $\mathbf{Q}_{\beta1} = (\pi/5, \pi/5)$ and $\mathbf{Q}_{\beta2} = (-\pi/5, \pi/5)$ as shown in the inset of Supplementary Fig. 10m. For the ordering vectors, the interaction tensors $\Gamma_{Q_{\nu}}^{ij}$ to satisfy the four-fold rotational symmetry are given by $\Gamma_{\alpha1}^{yy} = \Gamma_{\alpha2}^{xx} = \gamma_1$, $\Gamma_{\alpha1}^{xx} = \Gamma_{\alpha2}^{yy} = \gamma_2$, $\Gamma_{\alpha1}^{zz} = \Gamma_{\alpha2}^{zz} = \gamma_3$, $\Gamma_{\beta1}^{xx} = \Gamma_{\beta1}^{yy} = \Gamma_{\beta2}^{xx} = \Gamma_{\beta2}^{yy} = \gamma_4$, $-\Gamma_{\beta1}^{xy} = -\Gamma_{\beta1}^{yx} = \Gamma_{\beta2}^{xy} = \Gamma_{\beta2}^{yx} = \gamma_5$, $\Gamma_{\beta1}^{zz} = \Gamma_{\beta2}^{zz} = \gamma_6$ (the others are zero), where x = [100], y = [010], and z = [001]. We set $\hat{J} = 1$ as the energy unit of the model, and introduced the anisotropic parameters $\gamma_1 = 0.85\gamma_3$, $\gamma_2 = 0.95\gamma_1\gamma_3$, $\gamma_4 = 0.9\kappa\gamma_3$, $\gamma_5 = 0.025\kappa\gamma_3$, $\gamma_6 = \kappa\gamma_3$ for $\gamma_3 = 1$ and $\kappa = 0.95$. The parameters γ_1 and γ_2 (γ_4 and γ_5) stand for the in-plane bond-dependent anisotropy that fix the spiral plane, while γ_3 (γ_6) denotes the easy-axis anisotropy that favors the SkL at $\mathbf{Q}_{\alpha1}$ and $\mathbf{Q}_{\alpha2}$ ($\mathbf{Q}_{\beta1}$ and $\mathbf{Q}_{\beta2}$) [S12]. We set the parameters so as to satisfy $\chi_{Q_{\alpha1}} = \chi_{Q_{\alpha2}} > \chi_{Q_{\beta1}} = \chi_{Q_{\beta2}}$ by taking $\kappa = 0.95$, which means that the helix with $\mathbf{Q}_{\alpha1}$ or $\mathbf{Q}_{\alpha2}$ has smaller energy than that with $\mathbf{Q}_{\beta1}$ and $\mathbf{Q}_{\beta2}$. It is noted that the anisotropic parameters γ_1-γ_6 are related to the structure of the Fermi surfaces in addition to the spin-orbit coupling, but we here regard them as phenomenological parameters. The last term of Eq. (S7) represents the Zeeman term under an external magnetic field H along the [001] direction. The energy in Eq. (S7) is minimized by performing simulated annealing from high temperature. The simulations are carried out with the standard Metropolis local updates. The results are obtained for systems with $N = 100^2$ sites under periodic boundary conditions. In each simulation, we independently performed simulated annealing for different H to find low-energy spin configuration by gradually reducing the temperature with the rate $T_{n+1} = aT_n$ where T_n is the temperature in the nth step. We take $a = 0.99995 - 0.99999$ and the final temperature is typically taken at $T = 0.01$, which is reached by performing a total

of 10^5-10^6 Monte Carlo sweeps.

Supplementary Fig. 10 shows the magnetic field dependence of stable spin textures theoretically calculated for $\mathbf{H} \parallel [001]$ with $\hat{K} = 0$ and $\kappa = 0.95$. We have mainly identified four distinct magnetic phases (I, II, III, and IV) before reaching the forced ferromagnetic (FM) phase. The simulated real-space distribution of local magnetization $\mathbf{m(r)}$ and the corresponding momentum-space distribution of the spin structure factor $|\hat{\mathbf{m}}(\mathbf{Q}_\nu)|^2$ for each phase are displayed in Supplementary Fig. 10a-e and f-j, respectively. The zero-field state, phase I, is a single-Q state hosting a screw spin texture (Supplementary Fig. 10a). By increasing H, the double-Q state without four-fold symmetry is stabilized in phase II, which shows the rhombic SkL spin texture (Supplementary Fig. 10b). In phase III, the spin texture is characterized by the four-fold-symmetric double-Q structure corresponding to the square SkL state (Supplementary Fig. 10c). Further increase of H drives the transition into phase IV characterized by the double-Q modulation consisting of spin vortices (Supplementary Fig. 10d,e). In phase IV, the intensity distribution of spin structure factor breaks (holds) the four-fold symmetry at lower-H (higher-H) region, reflecting the presence (absence) of tiny $\hat{m}_{[001]}(\mathbf{Q}_{\alpha 2})$ component. Supplementary Fig. 10k-o shows the H dependence of the magnetization, scalar spin chirality and individual $|\hat{\mathbf{m}}(\mathbf{Q}_\nu)|^2$ components. The above simulation well reproduces the observed H-induced magnetic phase transitions I→II→III→IV→FM (Fig. 2 in the main text).

Note that the present theoretical simulation results also explain several detailed features in the neutron/X-ray scattering pattern. For phase II, the theory predicts much larger $|\hat{\mathbf{m}}(\mathbf{Q}_{\alpha 2})|^2$ than $|\hat{\mathbf{m}}(\mathbf{Q}_{\alpha 1})|^2$, in consistent with the experimental observation of larger magnetic scattering intensity for $\mathbf{Q}_1$+$\mathbf{Q}_2$ reflection than $\mathbf{Q}_1$-$\mathbf{Q}_2$ reflection in Supplementary Figs. 4b, e, and h. For phase IV, $|\hat{\mathbf{m}}(\mathbf{Q}_{\alpha 1})|^2$ and $|\hat{\mathbf{m}}(\mathbf{Q}_{\alpha 2})|^2$ are predicted to be very small, which explains the observed weakness of $\mathbf{Q}_1$+$\mathbf{Q}_2$ reflection in the experiment (Fig. 2d in the main text and Supplementary Fig. 8). The simulation suggests that the vortex-lattice spin state with and without four-fold symmetry (Supplementary Figs. 10d and e) are energetically almost degenerated in the phase IV, accounting for the experimental appearance of these two states in Supplementary Fig. 8.

While further fine tuning of the magnetic anisotropy and/or κ value may be required to fully reproduce all the details of observed phase transitions, our main conclusion here, i.e., frustration between the distinct χ_q peaks ($\chi_{Q_{\beta 1}}/\chi_{Q_{\alpha 1}} \sim 1$) stabilizes the two distinct SkL states

under the easy-axis magnetic anisotropy, will be valid in general.

X. Charge density wave and crystal structural transition

$EuAl_4$ has been reported to host a charge density wave characterized by the incommensurate ordering vector along the [001] axis below 145 K [S10]. Because the sinusoidal wave is generally centrosymmetric, there must be a point where the inversion center of the original crystal structure coincides with that of the sinusoidal wave in case of the incommensurate order. Therefore, the present system can be considered as centrosymmetric even with the incommensurate charge density wave.

In Ref. [S10], tiny orthorhombic distortion of crystal structure from the high-temperature tetragonal phase has also been reported below 12.2 K at 0 T. Since the system exhibits the incommensurate magnetic modulation in both tetragonal (phases I & V) and orthorhombic (phases VI & IV) crystal structures (Supplementary Fig. 9), the reported small orthorhombic crystal distortion can be excluded as the origin of incommensurate magnetic modulation.

References

S1. Blume, M. in Resonant Anomalous X-Ray Scattering (eds Materlik, G., Sparks, C. J. and Fischer, K.) 495–512 (Elsevier, 1994).

S2. Neubauer, A. *et al*., Topological Hall Effect in the A Phase of MnSi, *Phys. Rev. Lett.* **102**, 186602 (2009).

S3. Nagaosa, N. *et al.* Anomalous Hall effect, *Rev. Mod. Phys.* **82**, 1539 (2010).

S4. Yasui, Y. *et al.* Imaging the coupling between itinerant electrons and localised moments in the centrosymmetric skyrmion magnet $GdRu_2Si_2$. *Nat. Commun.* **11**, 5925 (2020).

S5. Lux, F. R. *et al.* Chiral Hall Effect in Noncollinear Magnets from a Cyclic Cohomology Approach. *Phys. Rev. Lett.* **124**, 096602 (2020).

S6. Kurumaji, T. *et al.* Skyrmion lattice with a giant topological Hall effect in a frustrated triangular-lattice magnet. *Science* **365**, 914-918 (2019).

S7. Hirschberger, M. *et al.* Skyrmion phase and competing magnetic orders on a breathing Kagomé lattice. *Nat. Commun.* **10**, 5831 (2019).

S8. Kaneko, K. *et al.* Charge-Density-Wave Order and Multiple Magnetic Transitions in Divalent Europium Compound $EuAl_4$. *J. Phys. Soc. Jpn.* **90**, 064704 (2021).

S9. Wang, Z., Su, Y., Lin, S.-Z., & Batista, C. D., Meron, skyrmion, and vortex crystals in centrosymmetric tetragonal magnets. *Phys. Rev. B* **103**, 104408 (2021).

S10. Shimomura, S. *et al.* Lattice Modulation and Structural Phase Transition in the Antiferromagnet $EuAl_4$. *J. Phys. Soc. Jpn.* **88**, 014602 (2019).

S11. Hayami, S. and Motome, Y., Topological spin crystals by itinerant frustration. *J. Phys.: Condens. Matter* 33, 443001 (2021).

S12. Hayami, S. and Motome, Y., Square skyrmion crystal in centrosymmetric itinerant magnets. *Phys. Rev. B* 103, 024439 (2021).

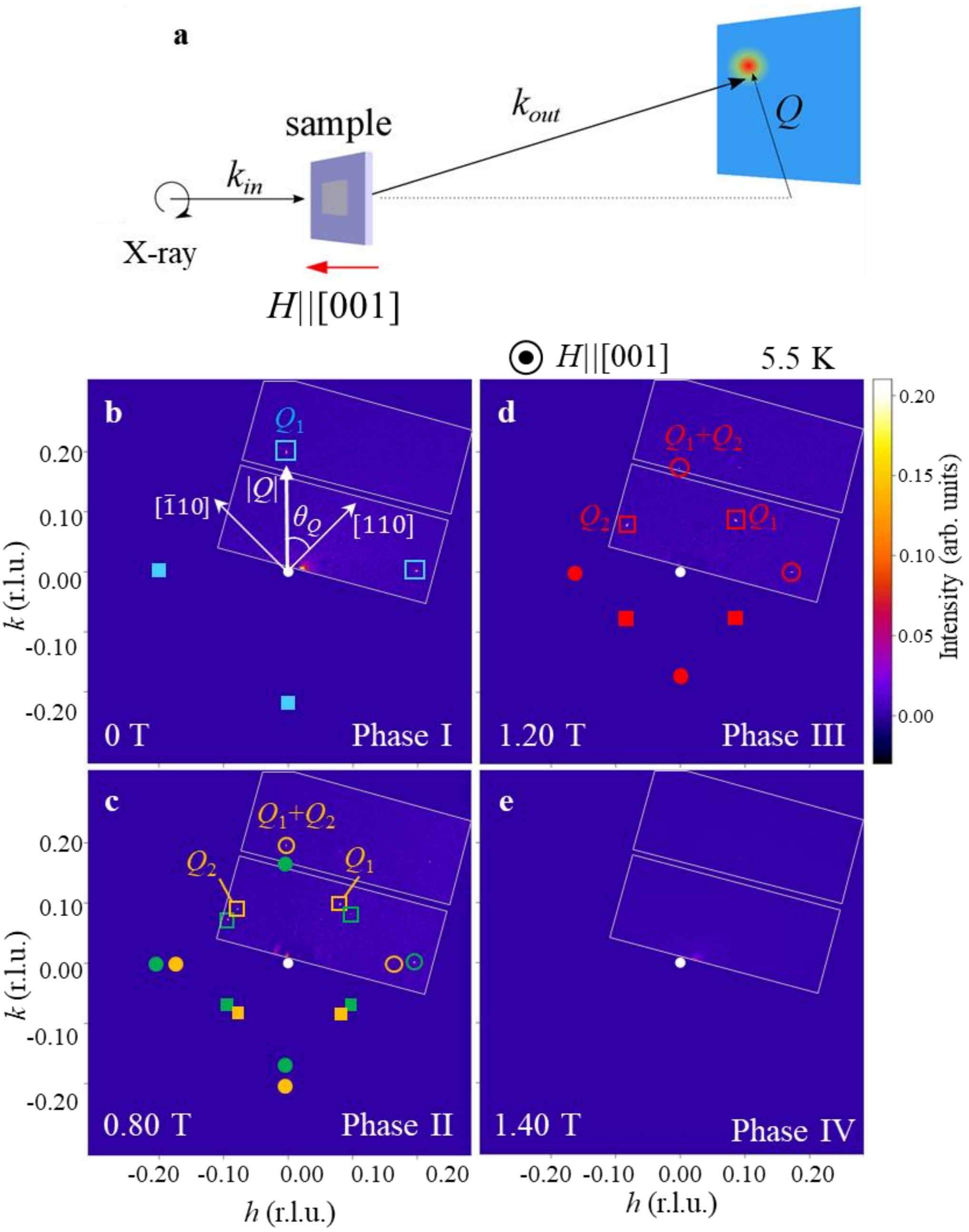

Supplementary Figure 1 | Magnetic field dependence of small-angle resonant elastic X-ray scattering patterns for $EuAl_4$. a, Schematic illustration of the experimental geometry for X-ray measurement. k_{in} and k_{out} are the incident and scattered X-ray wave vectors, respectively. **b-e**, Typical scattering patterns taken at 5.5 K with various magnitudes of magnetic field for H || [001]. The color scale indicates the scattering intensity. The white squares outline the area experimentally measured in the present setup. Open (filled) squares and circles indicate the observed (expected) spots.

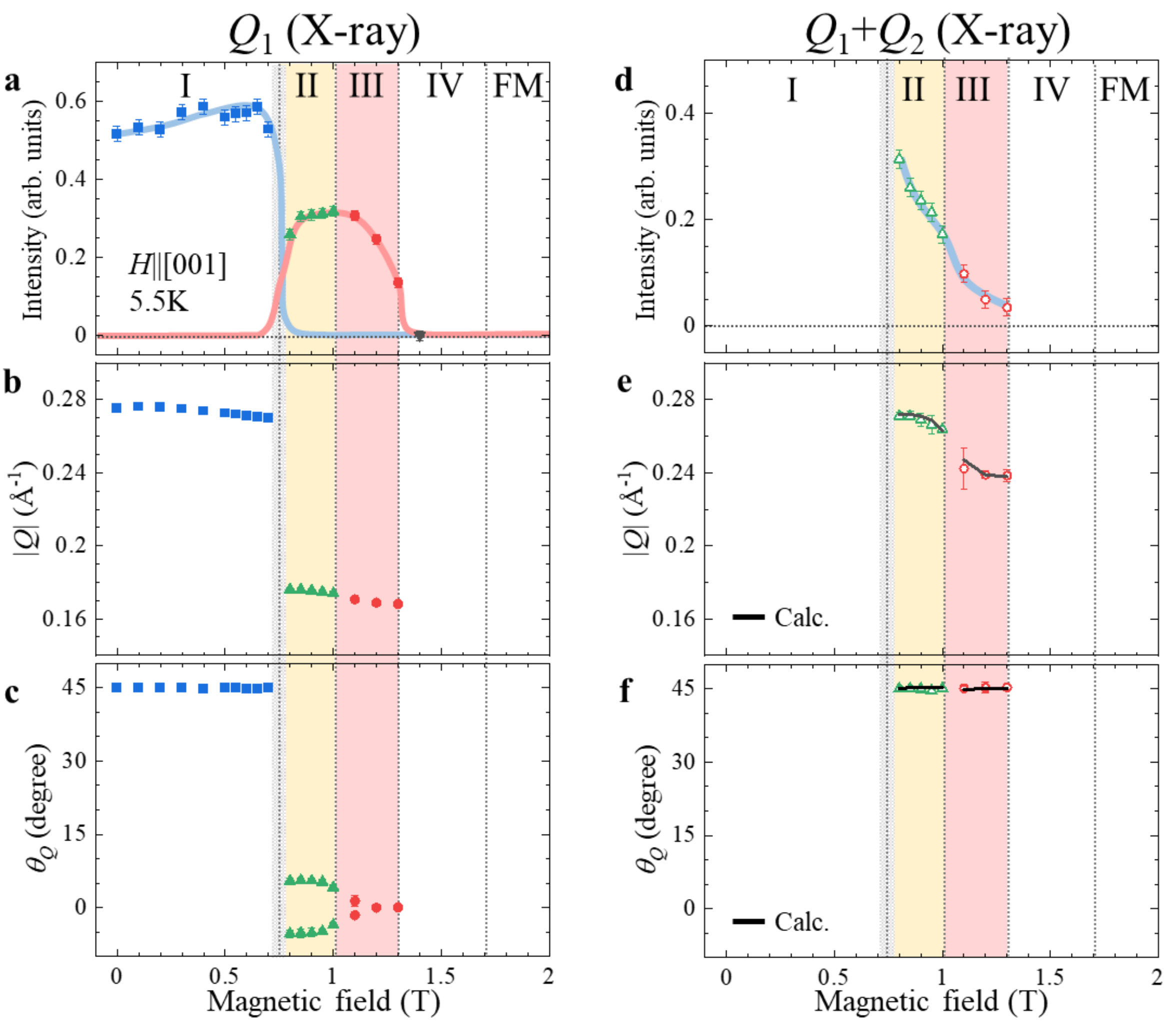

Supplementary Figure 2 │Magnetic-field dependence of the magnetic modulation vectors determined by small-angle X-ray scattering experiments. a, The integrated intensity of first-order fundamental magnetic reflection $\mathbf{Q}_1$ as a function of magnetic field for H || [001] at 5.5 K. (**b,c**) Magnetic field dependence of the magnitude $|Q|$ (**b**) and azimuth angle θ_Q (**c**) of the magnetic modulation vector $\mathbf{Q}_1$ in the (hk0) plane. Here, θ_Q is defined as the angle between the **Q**-direction and the [110] axis, as shown in Supplementary Fig. 1b. **d-f,** The corresponding data for the higher-order $\mathbf{Q}_1$+$\mathbf{Q}_2$ magnetic satellite reflection. The black curves in (**e,f**) represent the theoretical $|Q|$ and θ_Q values for $\mathbf{Q}_1$+$\mathbf{Q}_2$ reflection calculated from fundamental magnetic reflections in (**b,c**), which agree well with the experimental data. The gray regions between phases I and II indicate the phase coexistence region. The error bars are obtained from least-square fits of the peak profiles.

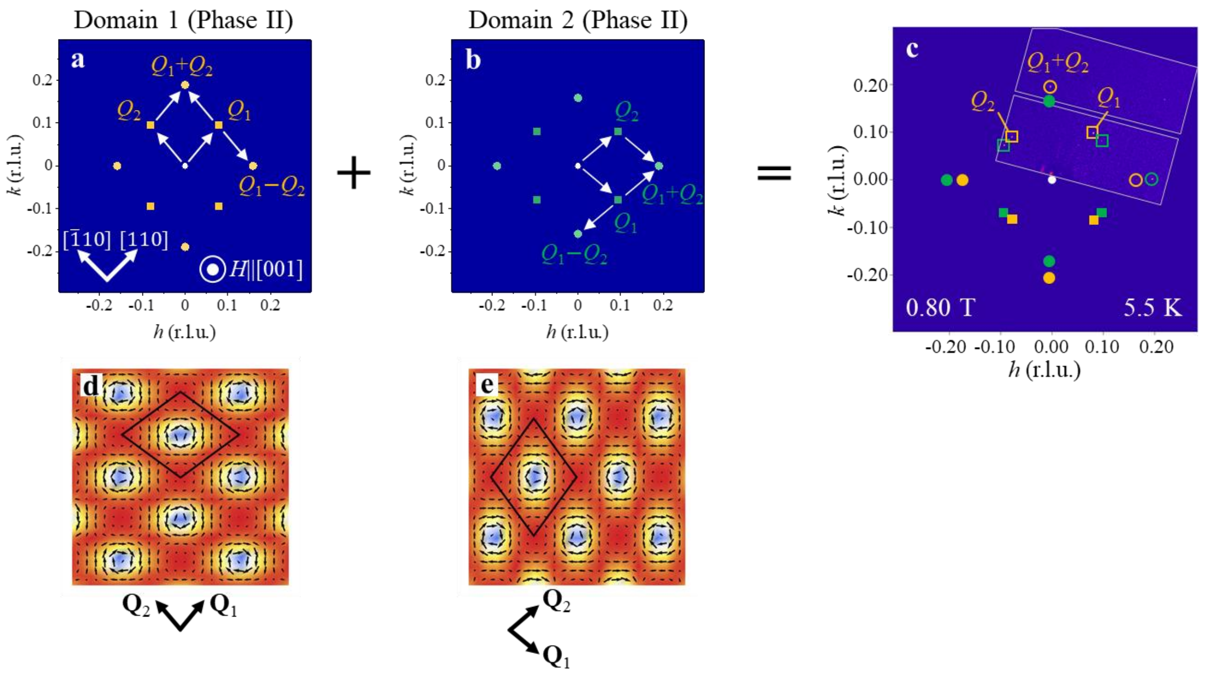

Supplementary Figure 3 │Magnetic domains in phase II. a,b, Reciprocal-space scattering patterns for two distinctive magnetic domains in phase II. **c**, Experimentally observed X-ray scattering pattern in phase II, showing the appearance of eight fundamental magnetic reflection spots. **d,e**, The real-space spin texture corresponding to the reciprocal-space scattering pattern in (**a,b**), calculated based on Eq. (2) in the main text.

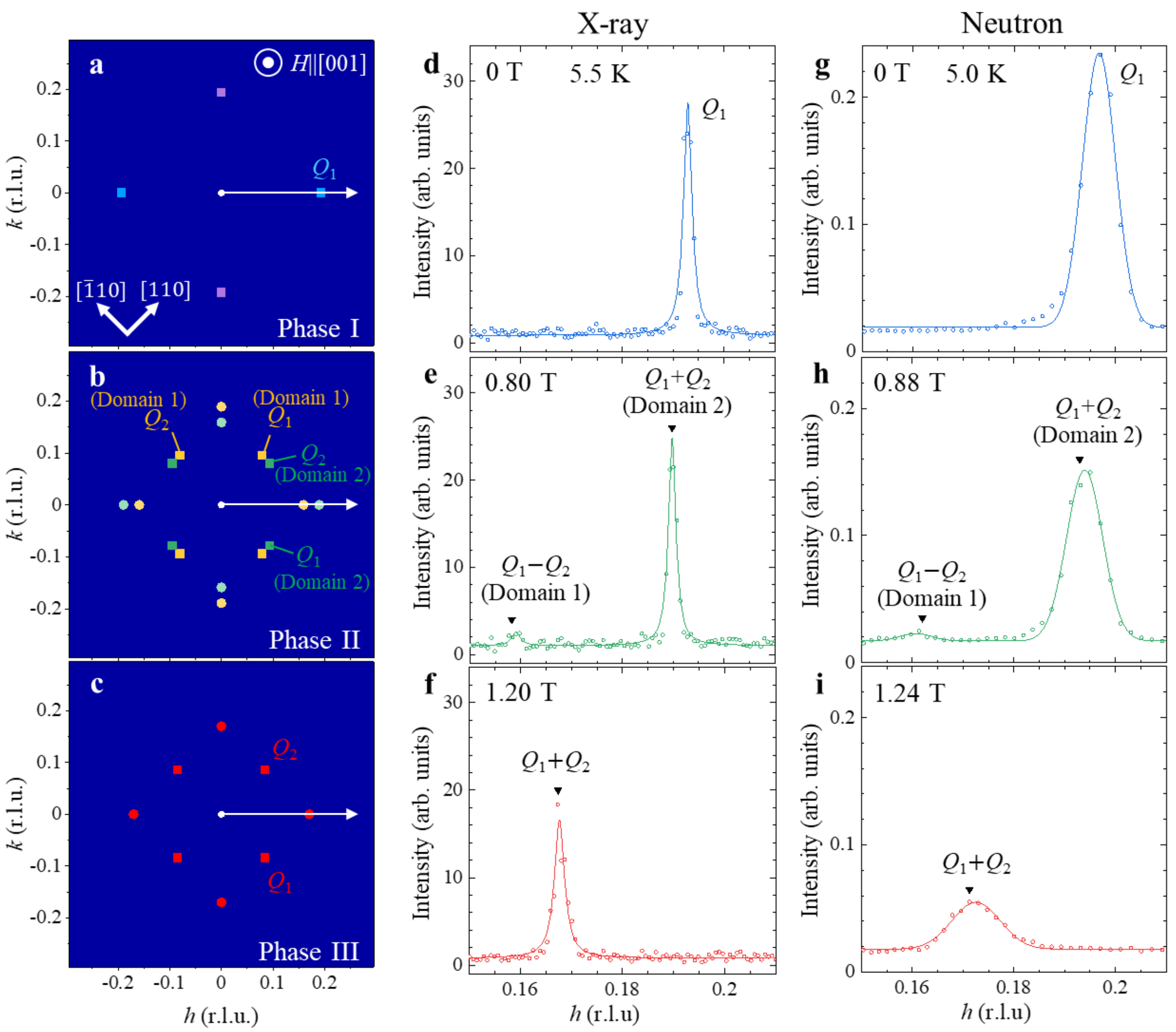

Supplementary Figure 4 │a-c, Schematic illustrations of reciprocal-space scattering pattern for each phase. **d-i**, Line-scan profiles along the [100] direction (corresponding to white arrows in (**a-c**)) for each phase, obtained based on the resonant X-ray scattering (**d-f**) and SANS (**g-i**) experiments. The black triangles in (**e,f,h,i**) represent the theoretical |*Q*| values for Q_1+Q_2 and Q_1-Q_2 reflections calculated from the fundamental magnetic reflections, which agree well with the observed peak positions.

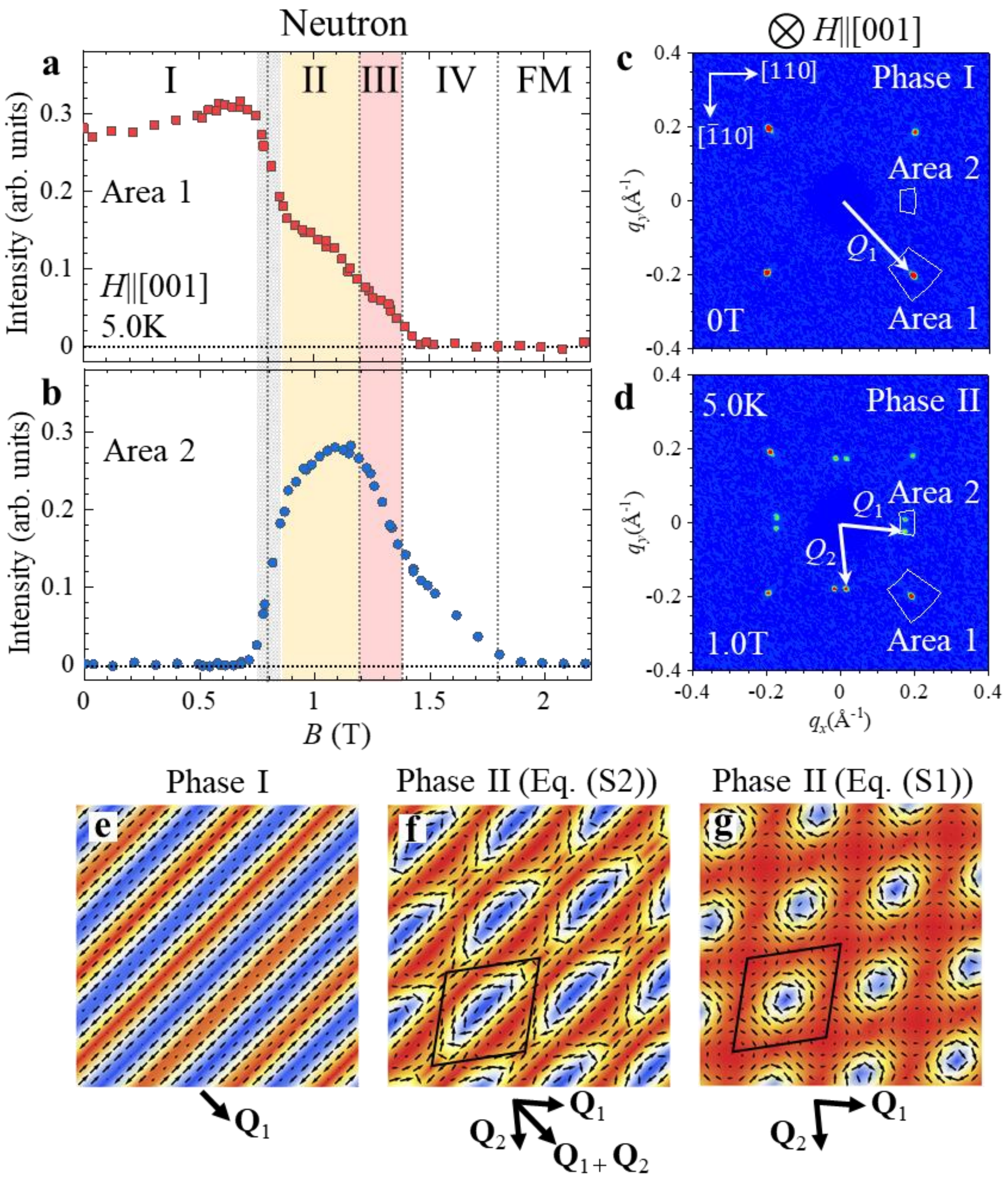

Supplementary Figure 5 │a,b, Magnetic-field dependence of the SANS intensities for Area 1 (**a**) and Area 2 (**b**) defined in (**c,d**). **e-g**, Schematic illustration of the screw spin texture in phase I (**e**), and rhombic skyrmion lattice in phase II calculated based on either Eq. (S2) (**f**) or Eq. (S1) (**g**). Background color represents the out-of-plane component of local magnetic moment m_z.

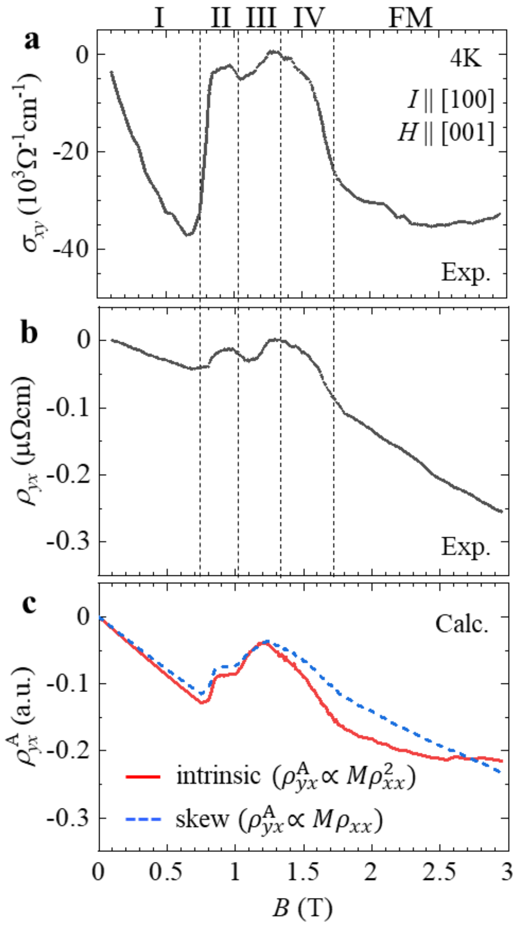

Supplementary Figure 6 │ Hall resistivity and Hall conductivity profiles for $EuAl_4$. **a,b**, Magnetic-field dependence of Hall conductivity σ_{xy} (a) and Hall resistivity ρ_{yx} (b) measured at 4 K. The Hall conductivity was calculated as $\sigma_{xy} = \rho_{yx}/(\rho_{xx}{}^2 + \rho_{yx}{}^2)$. **c**, Calculated anomalous Hall term ρ_{yx}^{A} originating from the intrinsic and skew scattering mechanism.

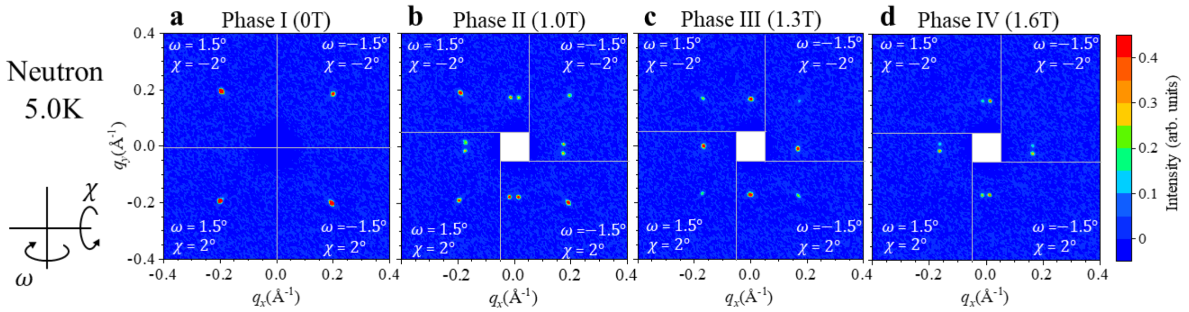

Supplementary Figure 7 │ a-d, The SANS patterns at 5 K in Fig. 2a-d in the main text were obtained by combining the four distinctive images measured at each rotating (ω) and tilting (χ) angles.

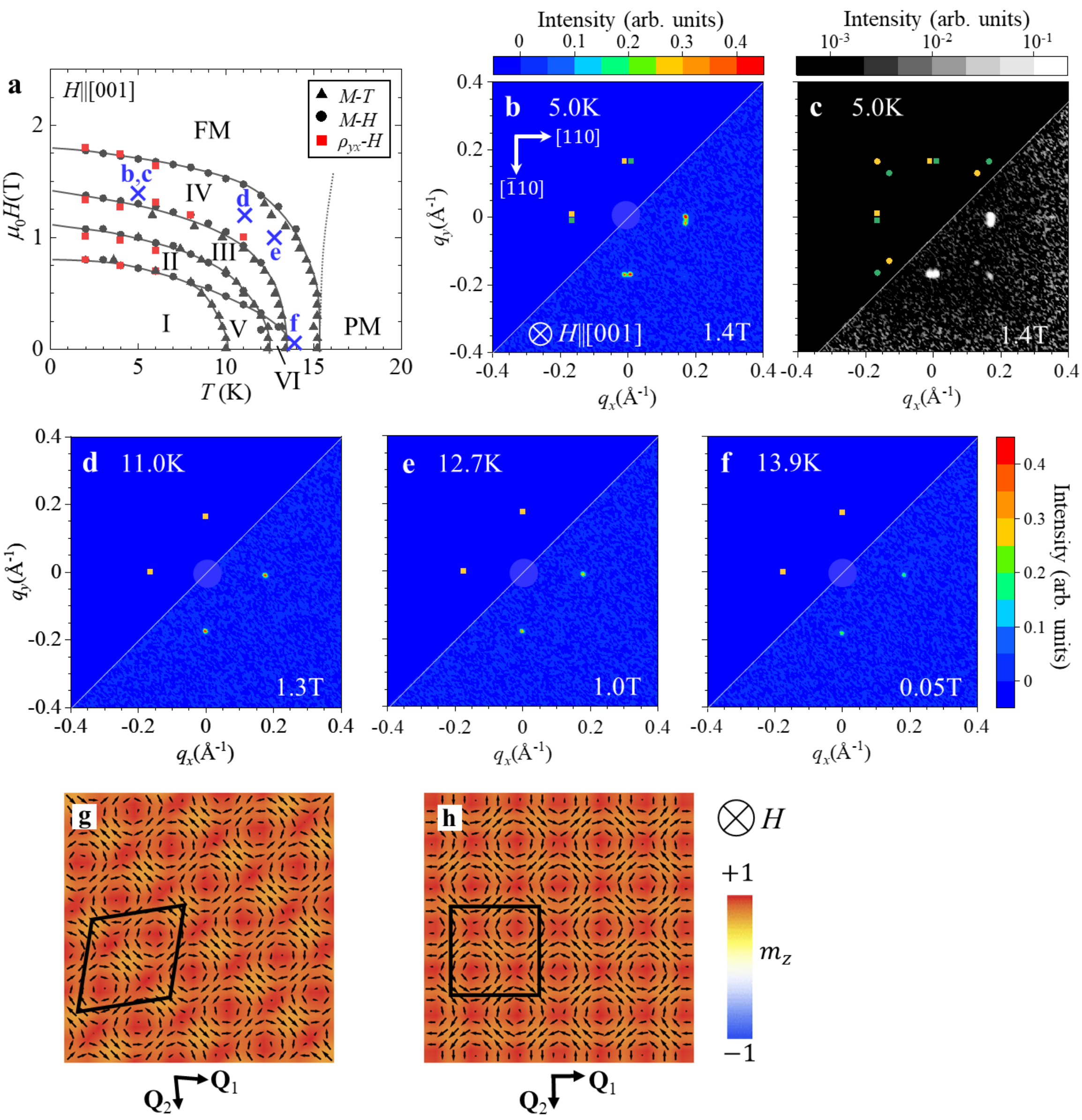

Supplementary Figure 8 │SANS patterns obtained for various temperature and magnetic field values in phase IV. a, Magnetic field(H)-temperature(T) phase diagram for **H** || [001]. **b-f**, Diffraction patterns taken at various T and H values indicated by the cross marks in (**a**). Lower right part shows the measured diffraction pattern and upper left part is the illustration of scattering pattern. Here, the data for b,c and d were measured in the field increasing process after zero field cooling at 5.0 K and 11.0 K, respectively. The data for e and f were obtained in the temperature increasing process at 1.0 T and 0.05 T, respectively. **g,h,** Rhombic and square vortex lattice spin texture described by Eq. (S3), which correspond to the low-T and high-T region of phase IV.

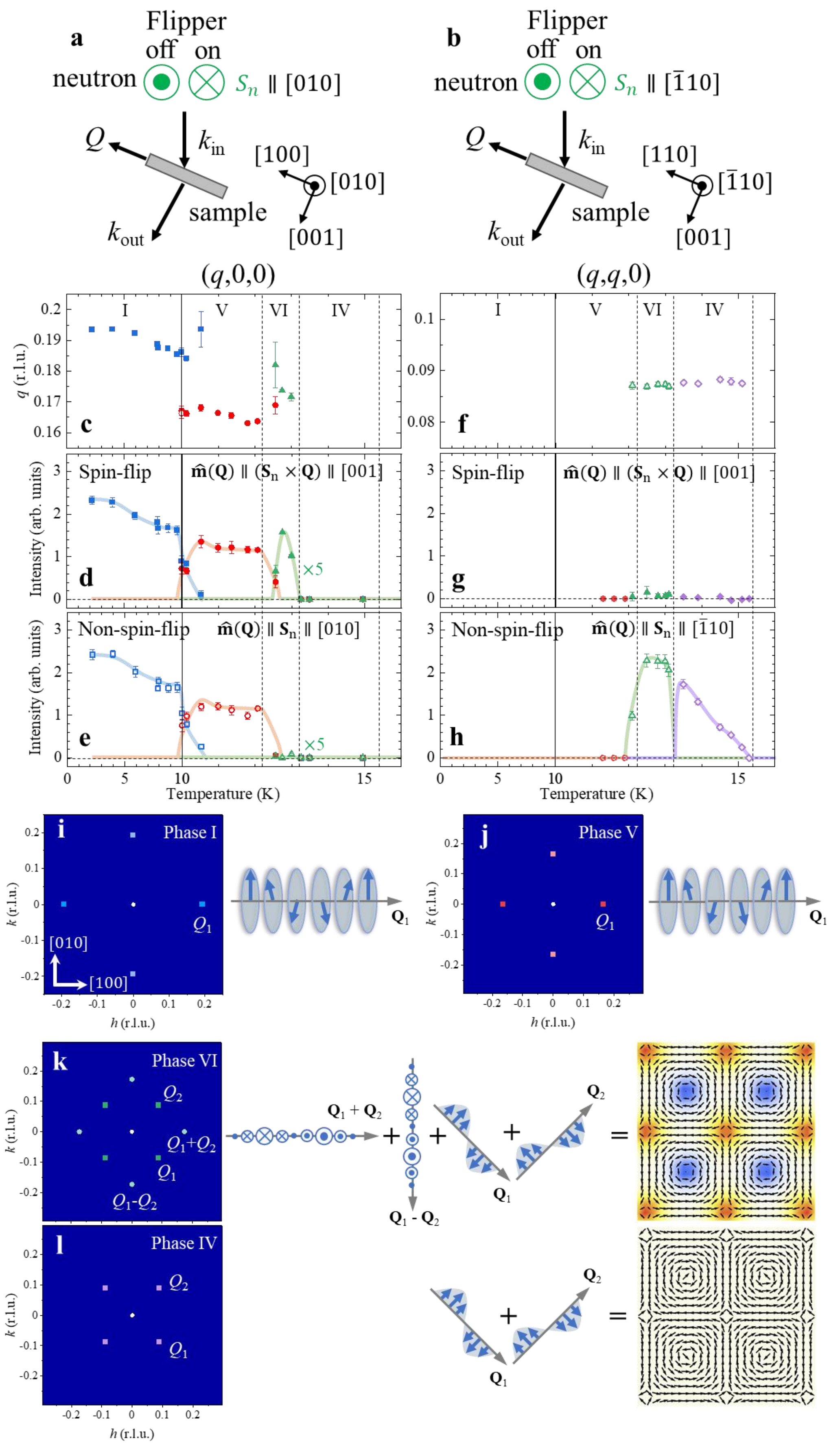
a
Flipper
off on
neutron
S_n ∥ [010]
Q
k_{in}
k_{out}
sample
[100]
[010]
[001]
b
S_n ∥ [$\bar{1}$10]
[110]
[$\bar{1}$10]
(q,0,0)
(q,q,0)
I
V
VI
IV
q (r.l.u.)
Spin-flip
m̂(Q) ∥ (Sn × Q) ∥ [001]
Non-spin-flip
m̂(Q) ∥ Sn ∥ [010]
m̂(Q) ∥ Sn ∥ [$\bar{1}$10]
×5
Intensity (arb. units)
Temperature (K)
Phase I
Phase V
Phase VI
Phase IV
Q_1
Q_2
Q_1+Q_2
Q_1-Q_2
$\mathbf{Q}_1 + \mathbf{Q}_2$
$\mathbf{Q}_1 - \mathbf{Q}_2$
k (r.l.u.)
h (r.l.u.)

Supplementary Figure 9 │a,**b**, Schematic illustration of polarized neutron scattering measurement setup for (**a**) (q, 0, 0) and (**b**) (q, q, 0) magnetic scattering. S_n represents the direction of the neutron polarization. **c-h**, Temperature dependence of the q value and the integrated intensity for SF and NSF scatterings for the (q, 0, 0) (**c**-**e**) and (q, q, 0) peaks (**f**-**h**). **i-l**, Schematic neutron diffraction patterns and the corresponding spin textures for phases I, V, VI, and IV. The error bars in **c**-**h** are obtained from least-square fits of the line scan profiles.

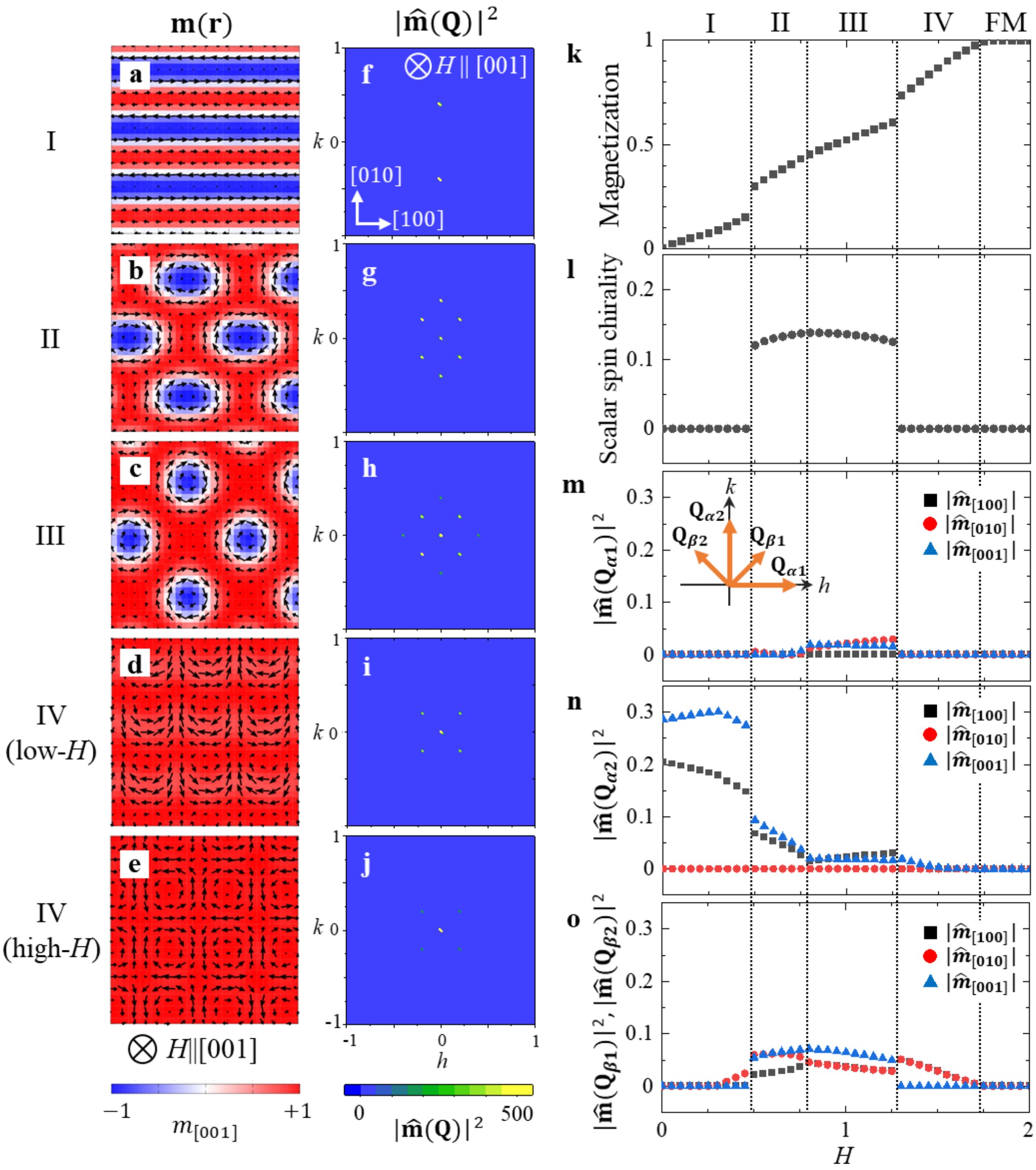

Supplementary Figure 10 | Theoretical simulation of spin textures and magnetic phase diagram. a-e, The distribution of local magnetization $\mathbf{m}(\mathbf{r})$ obtained by simulated annealing based on the model described by Eq. (S7). The background color represents the amplitude of $m_{[001]}$. The arrows indicate the in-plane component of $\mathbf{m}(\mathbf{r})$, and their lengths in **d** and **e** are multiplied by 1.5 and 2 compared with the ones in **a-c**, respectively. **f-j**, Simulated distribution of spin structure factor $|\hat{\mathbf{m}}(\mathbf{Q}_\nu)|^2$ in the reciprocal space, corresponding to (**a-e**). The color indicates $|\hat{\mathbf{m}}(\mathbf{Q}_\nu)|^2$ in the arbitrary unit. **k-o**, Magnetic field dependence of magnetization (**k**), scalar spin chirality (**l**), and modulated spin component $|\hat{\mathbf{m}}(\mathbf{Q}_\nu)|^2$ (**m-o**), theoretically calculated by simulated annealing for H || [001].
